# From mechanotransduction to manual therapy: advances in piezo/TRP channels and lumbar degeneration

**DOI:** 10.3389/fphys.2026.1853712

**Published:** 2026-07-22

**Authors:** Zhennan Liu, Tianzhong Peng, Wenli Li, Manhua Zhu

**Affiliations:** 1 Department of Rehabilitation Medicine, Nanchang Hongdu Hospital of Traditional Chinese Medicine, Nanchang, China; 2 Graduate School, Jiangxi University of Traditional Chinese Medicine, Nanchang, China

**Keywords:** lumbar degenerative disease, manual therapy, mechanotransduction, piezo ion channels, review, TRP channels

## Abstract

Degenerative lumbar spondylolisthesis and related lumbar instability involve more than segmental biomechanical imbalance. They also involve disturbed mechanotransduction mediated by mechanosensitive ion channels. This review brings together basic, mechanobiological, and clinical studies on Piezo1, Piezo2, and TRPV4 in the intervertebral disc, cartilage endplate, facet joint, ligamentum flavum, and sensory afferent system, and considers how manual therapy might affect segmental kinematics, load transfer, and the local microenvironment. Piezo1 is most often linked to load-driven amplification of inflammation, senescence, apoptosis, and fibrosis; Piezo2 is more closely tied to proprioceptive disturbance and mechanically evoked pain sensitization; and TRPV4 shows context-dependent effects shaped by loading pattern, osmotic milieu, and tissue state. Manual therapy may therefore influence Piezo/TRP-related mechanotransduction indirectly through changes in segmental motion, load distribution, and sensorimotor control, although direct evidence remains limited. The literature fits better with viewing manual therapy as a way of reshaping the mechanical microenvironment than as a molecular intervention that directly targets ion channels. Future work should test this working mechanistic model by linking standardized mechanical inputs to imaging, molecular, physiological, and symptom-based outcomes across the pathway from altered mechanics to biological responses and clinical benefit.

## 1 Search strategy and evidence integration

This review was designed as a structured mechanistic review rather than a formal systematic review. We searched PubMed/MEDLINE, Web of Science Core Collection, Scopus, and Embase from database inception to March 2026. Search terms included Piezo1, Piezo2, TRPV4, mechanotransduction, intervertebral disc, nucleus pulposus, annulus fibrosus, cartilage endplate, facet joint, ligamentum flavum, dorsal root ganglion, lumbar degeneration, degenerative lumbar spondylolisthesis, manual therapy, spinal manipulation, mobilization, traction, and relevant combinations. Reference lists of key articles and related reviews were also screened manually to capture early biomechanical studies, foundational work on mechanosensitive channels, and important methodological papers.

After duplicate removal, title and abstract screening, and full-text assessment, 199 publications were included in the narrative evidence synthesis and cited in the main text. During study selection, priority was given to human studies directly addressing DLS or lumbar degeneration. This was followed by experimental, translational, biomechanical, and clinical studies on the intervertebral disc, cartilage endplate, facet joint, ligamentum flavum, dorsal root ganglion, and manual therapy for low back pain. When direct lumbar evidence was limited, mechanistic studies from related musculoskeletal tissues or preclinical models were used only as supporting evidence. Conference abstracts, editorials, duplicate publications, and studies without a clear link to mechanotransduction, lumbar degenerative tissues, or manual therapy were excluded from further synthesis. Because the included studies differed substantially in model systems, tissue types, outcome measures, and intervention definitions, the evidence was integrated narratively rather than by quantitative meta-analysis. To avoid conflating direct DLS evidence with evidence from related degenerative conditions or preclinical mechanisms, the included literature was stratified by evidence source, relevance to DLS, and role in the argument of this review (**Table 1**).

**Table 1:** Evidence hierarchy and DLS relevance of the main evidence sources included in this review.

Evidence domain	Main evidence sources	DLS relevance	Role	References
DLS-specific clinical evidence	Imaging, epidemiology, clinical outcomes, segmental instability	Direct	Defines the DLS context	(Samuel et al., 2017; Rangwalla et al., 2023).
Lumbar degenerative tissue evidence	Disc, endplate, facet joint, ligamentum flavum, paraspinal tissues	Indirect to moderate	Supports tissue extrapolation	(Sun et al., 2020; Tong et al., 2023).
Preclinical mechanobiology evidence	Cells, animal models, ex vivo loading, organ-on-chip systems	Mechanistic	Supports biological plausibility	(Kim et al., 2021b; Cao et al., 2026).
Sensorimotor and pain-related evidence	DRG, sensory neurons, proprioception, inflammatory pain models	Indirect	Explains pain and sensory changes	(Woo et al., 2015; Obeidat et al., 2023).
Manual therapy and biomechanical evidence	Manipulation, mobilization, traction, biomechanical measurements, rehabilitation models	Limited DLS-specific	Supports mechanical-input modulation	(Jun et al., 2020; Langenfeld et al., 2025).

DLS, degenerative lumbar spondylolisthesis; DRG, dorsal root ganglion. DLS relevance indicates whether evidence directly supports DLS-specific conclusions or mainly provides mechanistic extrapolation.

## 2 Introduction

Degenerative lumbar spondylolisthesis (DLS) is a common spinal degenerative disorder in which a vertebral body shifts anteriorly or posteriorly after degeneration of the intervertebral disc, facet joints, and ligamentous structures, without a bony defect in the pars interarticularis (Rangwalla et al., 2023). Its prevalence rises with age: approximately 3%-8% in individuals younger than 40 years, 8%-23% in those aged 40–60 years, and as high as 23%-50% in those older than 60 years. Women are affected more often than men, with a reported sex ratio of approximately 1.3:1 to 6:1 (Wang et al., 2017). Samuel et al. (2017) argued that DLS often begins with disc degeneration and later involves the facet joints under conditions of chronic instability and increased stress, eventually producing vertebral slippage. L4-L5 is the segment most commonly affected, which fits its distinctive biomechanical setting (Köhli et al., 2024). The L5 vertebral body and transverse process are restrained by the iliolumbar ligament complex, giving L5-S1 substantial resistance to anterior translation; L4 lacks the same degree of peripheral ligamentous restraint and lies near the apex of lumbar lordosis, so it is exposed to greater sagittal shear during daily weight bearing (Cai et al., 2020; McGrath et al., 2022). As disc height falls because of dehydration and proteoglycan loss, axial load shifts toward posterior structures, especially the facet joints, which promotes facet degeneration, capsular laxity, and ligamentum flavum hypertrophy and eventually contributes to segmental instability and spondylolisthesis (Alonso et al., 2017). Because DLS is common, treatment is usually approached in a stratified way. Current guidelines still place conservative management first (Bydon et al., 2019). Manual therapy has therefore become a routine part of many conservative treatment programs because it is practical and relatively inexpensive (Chan et al., 2019; Kim et al., 2021a). Earlier mechanistic discussions focused mainly on neurophysiological regulation, but more recent work has moved down to the cellular and molecular levels (Bialosky et al., 2018).

Interest in mechanosensitive ion channels has grown because they sit at the interface between external mechanical stimulation and intracellular signaling. Sun et al. (2020) showed that aberrant mechanical stress activates Piezo1 and, through the Ca²^+^/NF-κB pathway, promotes NLRP3 inflammasome activation and IL-1β release in nucleus pulposus cells, thereby accelerating disc degeneration. Wang et al. (2021a) further found that increased matrix stiffness can also activate Piezo1, regulate oxidative stress-related senescence and apoptosis, and track positively with disc degeneration severity. Beyond the Piezo family, TRPV4 is widely expressed in disc cells and responds to osmotic change, mechanical stress, and inflammatory mediators (Kim et al., 2021b).

Viewed together, these findings place lumbar degeneration on two connected levels: segmental biomechanics and cell-level mechanotransduction. Manual therapy may alter the activation context of mechanosensitive channels by modulating the local mechanical environment, load transmission, and sensorimotor input. However, direct molecular regulation by manual therapy has not yet been demonstrated. The problem is that the evidence is still scattered. This review therefore examines the biological roles of Piezo and TRP channels in lumbar degeneration and considers how they might connect to mechanotransduction relevant to manual therapy. The novelty of this review lies in using DLS as a clinical entry point to extend research on mechanosensitive channels beyond general musculoskeletal degeneration. This framing supports a working mechanistic model that links segmental instability, tissue compartment-specific responses, and conservative intervention. By explicitly distinguishing direct evidence from extrapolated evidence, the review also defines a pathway for subsequent cross-scale validation.

## 3 Mechanobiological characteristics of piezo and TRP channels

Mechanosensitive ion channels are central executors of mechanotransduction. They turn physical stimuli into intracellular biochemical signals and are especially important in load-bearing tissues (Qin et al., 2021). Current evidence places Piezo1 closer to stress sensing and signal relay under high-load mechanical stimulation. It links membrane deformation to downstream biological responses and is often discussed as an integrator of mechanical information in degenerative settings (Lei et al., 2024). In annulus fibrosus cells, PIEZO1-mediated responses to cyclic stretch also influence BMP2-induced ossification programs, which suggests that Piezo1 does more than detect force; it also helps shape the transcriptional response that follows (Shitozawa et al., 2026). Piezo2 serves a different role, being mainly involved in touch, proprioception, and part of visceral sensory input, and is therefore more closely tied to mechanotransduction within sensory feedback pathways (Cheng et al., 2025).

TRPV4 behaves differently from Piezo channels. It is not a classic directly mechanically gated channel; instead, it responds to a combination of mechanical loading, osmotic change, and metabolic state, and it contributes to osteochondral homeostasis (Lyu et al., 2026). Pathogenic TRPV4 mutations markedly reshape the transcriptomic response of chondrocytes to mechanical loading, which means that TRPV4 is not simply reading stimulus magnitude. It also appears to matter for how stimulus quality is interpreted (Harissa et al., 2025). Under supraphysiological compression, TRPV4 can also drive COX2/PGE2-related inflammatory output (Cambria et al., 2021). That tissue-specific distribution of Piezo and TRP channels is likely to influence how mechanical signals are handled during degeneration and instability. Accordingly, this review summarizes evidence on Piezo channels and TRPV4 by tissue and system attribution, mechanical context, candidate channels, major biological responses, and evidence boundaries. This synthesis highlights both shared features and tissue-specific differences across lumbar structures (**Table 2**).

**Table 2:** Piezo/TRPV4-related mechanobiological signals in lumbar spine tissues.

Tissue/system	Mechanical context	Putative channels	Key responses	Evidence boundary
Nucleus pulposus/annulus fibrosus	Compression, stretch, osmotic stress, inflammation	Piezo1; TRPV4	Ca2+ influx, NF-κB/NLRP3, ROS, matrix remodeling	Supported mainly by disc degeneration models; indirect for DLS (Sun et al., 2020; Liu et al., 2023).
Cartilaginous endplate	Compression, impact, sclerosis	Piezo1; TRPV4	YAP/TEAD, NLRP3, mTOR, autophagy	Mostly preclinical and ex vivo evidence (Sun et al., 2021b; Cao et al., 2026).
Facet cartilage	Shear, compression, inflammation	Piezo1/2; TRPV4	NF-κB/MAPK, ferroptosis, matrix degradation, pain sensitization	Largely extrapolated from osteoarthritis (Lee et al., 2021; Ely et al., 2025).
Ligamentum flavum/facet capsule	Sustained stretch, stress concentration, stiffness increase	Piezo1	IL-6, TGF-β, WISP-1, profibrotic signaling	Direct channel evidence is limited (Liu et al., 2022; Tong et al., 2023).
Thoracolumbar fascia/paraspinal muscles	Stretch, shear, stiffness change, disuse	Piezo1; TRPV4	Matrix remodeling, mechanosensation, proprioceptive input	Not specific to DLS (Peng et al., 2022; Brandl et al., 2023).
DRG/sensory pathways	Compression, stretch, inflammatory sensitization	Piezo1; Piezo2	Mechanically evoked currents, hypersensitivity, altered proprioception	DLS-specific human evidence is insufficient (Murthy et al., 2018; Lee et al., 2024).

Putative channels reflect current mechanistic clues rather than direct causal evidence in human DLS.

### 3.1 Expression and functional contexts of Piezo and TRP in lumbar tissues

#### 3.1.1 Intervertebral disc

The intervertebral disc is an active load-bearing tissue, not a passive spacer, and both its development and its homeostasis depend on sustained mechanical regulation (He et al., 2026). In annulus fibrosus cells, different channels support different responses. Excessive stretch can activate the p38 MAPK pathway through TRPV4, inducing IL-6 and IL-8 transcription and PGE2 release and turning mechanical stimulation into inflammatory output (Cambria et al., 2020). Aberrant loading can also activate the Calpain2/BAX/Caspase3 cascade through Piezo1 and drive apoptosis (Liu et al., 2023). The first route fits inflammatory amplification more closely; the second speaks more to disturbed cell fate regulation. Organ-level studies likewise show that TRPV4 deficiency changes disc extracellular matrix composition and weakens tissue adaptation to sustained loading (Mark Kim et al., 2024). In that sense, TRPV4 appears to be involved in both homeostatic maintenance and pathological degeneration.

#### 3.1.2 Cartilage endplate

The cartilage endplate handles nutrient exchange, cushioning, and stress redistribution, so its integrity also depends on finely tuned mechanotransduction. Current evidence points to at least two parallel Piezo1-related pathways under compressive loading. One involves Ca²^+^ influx, YAP nuclear translocation, and YAP-TEAD complex formation, followed by activation of the NLRP3 inflammasome and upregulation of IL-1β and MMP13 (Cao et al., 2026). The other involves the Piezo1/P65 axis, increased NAT10 transcription, ac4C-mediated stabilization of mTOR mRNA, suppression of autophagy, and disruption of metabolic homeostasis (Sun et al., 2026). Even without obvious macrostructural damage, a single impact injury may trigger inflammation and metabolic dysfunction through Piezo1 and start the later course of disc degeneration (Sun et al., 2021b).

#### 3.1.3 Facet joints and articular cartilage

Facet joint cartilage is an important part of posterior column stability in the lumbar spine, and its degeneration may share mechanotransductive mechanisms with other load-bearing joints (Köhli et al., 2024). In articular cartilage, Piezo1-mediated mechanical responses run through several pathways. Excessive mechanical strain can induce Ca²^+^ influx, activate the calcineurin/NFAT1 axis, and drive chondrocyte apoptosis and matrix degradation (Ren et al., 2023). The same process intersects with NF-κB/MAPK signaling and ferroptosis pathways and is further amplified by inflammatory mediators such as IL-1α, TNF-α, and IL-17A (Chen et al., 2026). Chondrocyte-specific double knockout of Piezo1/2 attenuates structural damage and pain behavior in post-traumatic osteoarthritis (Ely et al., 2025), which suggests that mechanosensitive channels may contribute to both tissue degeneration and pain transduction. Whether lumbar facet joints follow the same mechanisms remains uncertain, but the available evidence is enough to keep them in the discussion.

#### 3.1.4 Fibrous stabilizing structures, including the ligamentum flavum and joint capsule

The ligamentum flavum and joint capsule work together to maintain posterior column stability, and abnormal remodeling in these tissues can weaken segmental restraint and worsen mechanical imbalance. Sustained mechanical stress activates the WISP-1/Hedgehog-Gli1 axis in the ligamentum flavum and drives fibroblasts toward a profibrotic phenotype (Sun et al., 2021a). Dysregulation of the miR-10396b-3p/IL-11 axis further promotes collagen synthesis and matrix deposition (Li et al., 2021). Mechanical stress can also upregulate periostin and connect inflammatory amplification with fibrotic remodeling through IL-6 expression (Yabu et al., 2022). At the same time, clusterin can be induced by combined mechanical stress and TGF-β1 and can downregulate α-SMA and COL1A2 by inhibiting ALK5/SMAD3 activation, which suggests that ligamentum flavum fibrosis is not a one-way amplification process and still includes endogenous braking mechanisms (Liu et al., 2022). Direct evidence for Piezo1 in the ligamentum flavum still comes mainly from ossification models, where stretch is accompanied by Piezo1 upregulation and activation of osteogenesis-related molecules (Tong et al., 2023). More broadly, the evidence base for ligamentum flavum hypertrophy still centers on TGF-β, MAPK, NF-κB, PI3K/AKT, non-coding RNAs, and ECM protein networks; mechanosensitive ion channels are not yet supported by an equally complete framework (Silwal et al., 2024). Mechanical stress is clearly involved, but whether Piezo1 or TRPV4 sits near the center of that process still needs more direct experimental evidence.

#### 3.1.5 Thoracolumbar fascia and paraspinal muscle system

The thoracolumbar fascia and paraspinal muscle system jointly contribute to posterior lumbosacral stability and local mechanical signal transmission. Numerical models indicate that the thoracolumbar fascia can transmit reaction forces between the thoracolumbar region and pelvis across different postures and activities. This force transmission varies with intra-abdominal pressure, paraspinal muscle pressure, and trunk muscle coordination (Bojairami and Driscoll, 2021; El-Monajjed and Driscoll, 2021). Ultrasound studies have documented thoracolumbar fascial deformation during trunk extension and deadlifting. In patients with myofascial low back pain, exploratory ultrasound studies have reported increased thoracolumbar fascial thickness, calcification, and shear-wave stiffness (Bubnov and Kalika, 2022; Brandl et al., 2023). Together, these findings define a tissue context of sustained stretch, shear stress, and altered stiffness in which mechanosensitive channels may be engaged.

The paraspinal muscles translate this mechanical background into segmental loading. Fatty infiltration, fibrosis, and functional insufficiency are common in low back pain and degenerative spinal disorders, although causality remains uncertain (Noonan and Brown, 2021). Musculoskeletal modeling suggests that multifidus status can influence estimates of lumbar loading and segmental force distribution (Wang et al., 2023c). Studies of DLS have also associated paraspinal fatty infiltration and changes in muscle cross-sectional area with vertebral slippage (Cao et al., 2023; Liu et al., 2024b). Paraspinal muscle degeneration may therefore affect local compression, shear, and strain distribution, making it relevant to the broader degenerative process of the lumbar spine.

Compared with other lumbar tissues, evidence for Piezo/TRP channels in the thoracolumbar fascia and paraspinal muscles remains largely indirect. TRPV4 can integrate ECM-derived mechanical signals with Ca²^+^ signaling and participate in collagen remodeling and matrix mechanical regulation (Ji and McCulloch, 2020). Piezo1 contributes to mechanosensing in muscle stem cells and responses to disuse-induced muscle atrophy (Hirata et al., 2022; Peng et al., 2022). It may also sense matrix alignment and regulate collagen synthesis in fibrotic models (Rashidi et al., 2025). In addition, the thoracolumbar fascia is innervated by nociceptive fibers, suggesting that abnormal stretch may alter peripheral sensory input (Sinhorim et al., 2021).

Short-term changes in spinal alignment, thoracolumbar fascial stiffness, and fascial organization after manual release or fascial manipulation suggest that the mechanical environment of the fascia and paraspinal muscles is modifiable (Brandl et al., 2021; Sánchez-Vera et al., 2025). Nevertheless, tissue-specific evidence does not yet show that these degenerative changes are directly driven by mechanosensitive channels. The thoracolumbar fascia and paraspinal muscle system therefore mainly help explain functional pain and myofascial phenotypes in DLS. Their molecular mechanisms still lack direct DLS-specific validation.

#### 3.1.6 Dorsal root ganglion and sensory afferent system

The dorsal root ganglion is one of the main sites where an abnormal mechanical environment becomes a symptom. In that setting, the relevance of mechanosensitive ion channels extends from tissue force sensing to neural coding. Piezo1 is functionally expressed in DRG neurons, which gives a plausible basis for its role in peripheral mechanical signal transduction (Roh et al., 2020). It is found more often in TRPV1-positive nociceptive neurons, and interventions targeting Piezo1 attenuate mechanically activated currents and reduce mechanical hypersensitivity in inflammatory states (Lee et al., 2024). Piezo2 is more clearly linked to low-threshold mechanosensation and proprioceptive encoding (Woo et al., 2015); after injury, it also contributes to touch-evoked pain and mechanical allodynia (Szczot et al., 2018; Xie et al., 2023). Other work shows that dynamic movement of small vessels within the DRG under nerve injury conditions can be sensed by Piezo2 on sensory neurons, triggering clustered firing and episodic spontaneous pain (Xie et al., 2025). Aberrant activation of mechanosensitive channels within the DRG is therefore likely to be an important step in the transition from pathological stress to clinical pain, although the full network of actions in lumbar degenerative disease still needs clarification.

### 3.2 Fundamental mechanisms of mechanotransduction and dependence on pathological thresholds

Across the intervertebral disc, cartilage endplate, facet joint cartilage, and sensory afferent system, Piezo/TRP channels can be understood as shared translators of mechanical information. They convert membrane stress, changes in cell-matrix coupling, and shifts in local fluid or osmotic conditions into Ca²^+^-dependent intracellular signals, which then engage the cytoskeleton, inflammatory networks, and matrix metabolism (Nims et al., 2024). The key question is not simply whether a cell is loaded, but how that load is interpreted and whether the downstream program favors homeostasis or injury. The process is not linear. Once the extracellular matrix is degraded, especially when the pericellular matrix is damaged, local stress transmission changes and later mechanical signals are amplified or rewritten (Nieuwstraten et al., 2025). In degenerative human nucleus pulposus cells, increased matrix stiffness can amplify mechanical stress through Piezo1 and induce Drp1-mediated mitochondrial fission, ROS accumulation, and apoptosis, indicating that changes in the mechanical microenvironment are both a consequence of degeneration and a driver of further aberrant transduction (Ke et al., 2023b). Channel output also depends on more than the simple presence of load; timing, duration, and loading configuration matter. Organ culture studies show that TRPV4 establishes different inflammatory networks under static and dynamic compression (Easson et al., 2023). At the same time, TRPV4 knockdown in nucleus pulposus cells suppresses autophagy and reduces ECM synthesis (Matsuo et al., 2025), which suggests that TRPV4 is involved both in injury under abnormal loading and in baseline homeostatic maintenance. This interpretation matches observations in other load-bearing tissues, where TRPV4 can preserve matrix and suppress inflammation in cartilage, and where annulus fibrosus cells respond differently to hydrostatic pressure and deviatoric strain (Fu et al., 2021; Taiji et al., 2024).

In short, the Piezo/TRP-mediated mechanotransduction network is highly context dependent. Within the physiological range, transmembrane calcium signaling supports adaptive tissue remodeling; once sustained overload, local stiffening, and related adverse microenvironments predominate, amplified channel signaling is more likely to push cells toward catabolism and degeneration (Li et al., 2025). Mechanosensitive ion channels therefore act both as force sensors and as regulators of tissue mechanical responses. Framing lumbar degeneration in these terms helps explain why biomechanical imbalance extends to the microscopic level and also gives a more specific basis for discussing the mechanical effects associated with manual therapy.

## 4 Roles of piezo/TRP channels in mechanical imbalance during lumbar degeneration

At the level of the motion segment, lumbar degeneration and instability are better understood as a progressive multitissue imbalance involving the intervertebral disc, cartilage endplate, and surrounding stabilizing structures under chronic abnormal stress, rather than as failure of a single structure in isolation (Fearing et al., 2018). As degeneration advances, intradiscal pressure falls across multiple directions of motion, indicating loss of the cushioning and load-sharing function normally provided by the nucleus pulposus and a corresponding shift in force transmission within the segment (Liebsch and Wilke, 2025). Abnormal loading is therefore not just a companion of degeneration. By reshaping the local microenvironment, it may also help drive progression. Piezo/TRP channels are likely part of that bridge between macroscopic mechanical imbalance and cellular or tissue-level pathology. The following sections review the evidence from that perspective.

### 4.1 Aberrant mechanotransduction in lumbar degeneration

**Figure 1:**
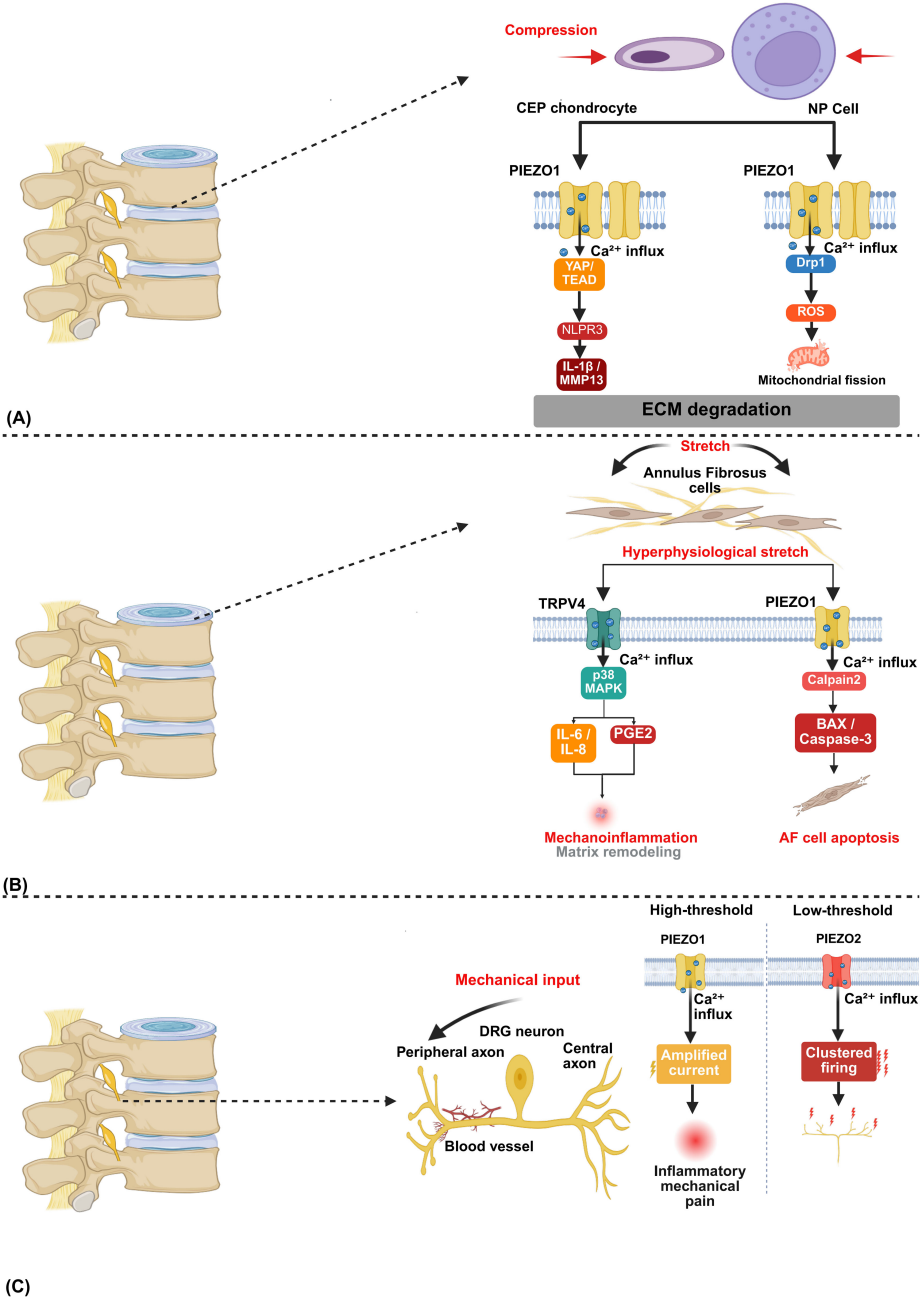
Schematic representation of Piezo/TRP channel-mediated mechanotransduction and downstream pathological outputs in lumbar degeneration-related tissues. **(A)** Under compressive loading, cartilage endplate cells and nucleus pulposus cells may undergo aberrant mechanotransduction centered on PIEZO1-mediated Ca²^+^ influx. On the endplate side, this process is associated with activation of the YAP/TEAD-NLRP3 axis and upregulation of IL-1β and MMP13; on the nucleus pulposus side, it is associated with Drp1-related mitochondrial fission, ROS accumulation, and ECM degradation. **(B)** Under supraphysiological tensile loading, annulus fibrosus cells may generate mechanical inflammatory responses via the TRPV4-p38 MAPK axis, including IL-6, IL-8, and PGE2, and may also undergo apoptosis via the PIEZO1-Calpain2-BAX/Caspase-3 axis. **(C)** Abnormal mechanical inputs generated by degenerative segments may also influence dorsal root ganglion neurons through sensory afferents; high-threshold mechanical stimuli are more likely to amplify mechanically activated currents and contribute to inflammatory mechanical pain via PIEZO1, whereas low-threshold inputs may participate in aberrant firing and mechanical sensitization via PIEZO2. This figure summarizes the pathways most strongly supported by current evidence; specific outputs remain dependent on loading pattern, exposure duration, and tissue state. CEP, cartilage endplate; NP, nucleus pulposus; AF, annulus fibrosus; DRG, dorsal root ganglion; ECM, extracellular matrix; ROS, reactive oxygen species. Created in BioRender. HA, Y. (2026) https://BioRender.com/5hzqit8.

Lumbar degeneration and instability develop through ongoing redistribution of load and should not be reduced to the simple sum of degeneration in several tissues. As degeneration advances, intradiscal pressure decreases across different directions of motion, showing that the buffering role once carried mainly by nucleus pulposus hydrostatic pressure is fading and that tissues are sensing and transmitting mechanical stimuli differently (Liebsch and Wilke, 2025). Experimental lumbar instability also produces pore-like remodeling of the cartilage endplate together with osteoclast recruitment, which suggests that abnormal loading is both a result of degeneration and a force that can push it further (Li et al., 2024). In this setting, Piezo and TRP family channels are best viewed as readout interfaces through which abnormal mechanical inputs enter cellular programs, converting cues such as pressure, tension, osmolarity, and matrix state into intracellular biochemical signals (Ke et al., 2025). For disc cells, mechanical stimulation is not automatically injurious; low-to-moderate compression, osmotic loading, or hydrostatic pressure is more compatible with homeostasis and matrix synthesis, whereas loading beyond the adaptive range more often shifts the response toward protease upregulation and catabolism (Fearing et al., 2018). Piezo1, in particular, is highly sensitive to membrane tension and membrane deformation and may play a larger part in the early sensing of high-strain perturbations (Zhao et al., 2018). Lumbar degeneration is therefore better understood as progressive metabolic disequilibrium produced by the interaction of abnormal stress and a changing local microenvironment, not as dysregulation of a single pathway. Different tissues also read compressive, tensile, and aberrant sensory inputs differently; the main pathways are summarized schematically in **Figures 1A–C**.

### 4.2 Piezo1-mediated amplification of degenerative injury

Among the channels discussed here, Piezo1 is the clearest candidate for amplifying abnormal mechanical input. In nucleus pulposus cells, mechanical stretch upregulates Piezo1 and activates the Ca²^+^/NF-κB/NLRP3 inflammatory axis, whereas Piezo1 silencing reverses this process. That makes Piezo1 more than a passive companion of degeneration; it places the channel upstream of pro-inflammatory programs triggered by abnormal loading (Sun et al., 2020). Additional studies show that increased matrix stiffness, mechanical overload, and disturbed mitochondrial dynamics can all amplify ROS accumulation, cellular senescence, and apoptosis through Piezo1, making short-lived mechanical events more likely to settle into persistent injury states (Shi et al., 2022; Ke et al., 2023b). The effect is not limited to the nucleus pulposus. In the cartilage endplate, Piezo1 promotes degeneration through several pathways: the Ca²^+^/CaMKII/Drp1 axis enhances mitochondrial fission and dysfunction and worsens senescence and apoptosis (Lin et al., 2024); Ca²^+^ influx together with F-actin remodeling promotes YAP activation and couples with NLRP3 inflammasome activation (Peng et al., 2025; Cao et al., 2026); and the Piezo1/P65 axis increases NAT10 expression, stabilizes mTOR mRNA through ac4C modification, suppresses autophagy, and disrupts metabolic homeostasis (Sun et al., 2026). These findings suggest that the cartilage endplate may be an active amplification interface through which abnormal load reaches deeper disc tissues. Piezo1-related injury has also been linked to disturbed iron homeostasis: in nucleus pulposus cells, mechanical overload can induce Piezo1-mediated iron influx, trigger GPX4 downregulation and lipid peroxidation, and ultimately promote ferroptosis (Xiang et al., 2024).

### 4.3 Piezo2, proprioceptive dysfunction, and pain transformation

Piezo2 is the main mechanotransduction channel in proprioceptive neurons. Its deletion impairs stretch-evoked discharge and leads to abnormal spinal alignment and reduced skeletal integrity, which suggests that distorted sensory feedback can itself reshape axial load regulation (Woo et al., 2015; Assaraf et al., 2020). Piezo2 also enters the picture during pain transition by contributing to mechanical sensitization. Its deletion significantly alleviates mechanically evoked hypersensitivity induced by inflammation or nerve injury (Murthy et al., 2018), whereas activation of the Epac1-Piezo2 signaling axis exacerbates mechanical hyperalgesia (Eijkelkamp et al., 2013). Osteoarthritis models further show that selective deletion of Piezo2 in nociceptors reduces mechanical sensitization and that these neurons largely overlap with the Ntrk1/TrkA lineage (Obeidat et al., 2023), pointing to a connection between Piezo2 and NGF-related mechanically evoked pain responses. Piezo1 also deserves mention here: it is functionally expressed in TRPV1-positive DRG nociceptors, shows regulatory coupling with TRPV1 (Roh et al., 2020), and reduces mechanical hypersensitivity when silenced in inflammatory settings (Lee et al., 2024). The broader picture is that Piezo family members may operate in parallel during peripheral sensitization and amplification of abnormal mechanical pain.

### 4.4 TRPV4 in load adaptation and degenerative progression

TRPV4 responds at the same time to loading pattern, osmotic pressure, and the state of cell-matrix coupling. Organ culture studies show that it activates different cytokine networks under static and dynamic compression, so a change in loading pattern can shift the inflammatory output (Easson et al., 2023). In sustained compression models, TRPV4 modulation has been linked both to promotion of matrix synthesis and to NF-κB activation with tissue degeneration, which means its effects depend heavily on stimulus duration and tissue state (Easson et al., 2022). *In vivo* studies add the same caution: conditional Trpv4 deletion does not simply yield an anti-degenerative phenotype, but changes disc extracellular matrix composition and remodels pathological responses of adjacent segments to abnormal stress (Mark Kim et al., 2024).

TRPV4 also changes its behavior with the surrounding microenvironment. Under mildly reduced hydrostatic pressure, the TRPV4 signaling axis helps maintain nucleus pulposus ECM homeostasis (Hu et al., 2025). Under hypo-osmotic conditions, TRPV4 expression and calcium signaling increase sharply, promoting release of pro-inflammatory mediators and accelerating inflammatory degeneration (Walter et al., 2016). That coupling is not entirely TRPV4-dependent, however, and additional pathways are involved (Sadowska et al., 2020). The relationship also runs in the opposite direction: inflammatory mediators can increase TRPV4 expression in human traumatic nucleus pulposus cells, further amplifying local mechanical sensitization and setting up positive feedback that drives degeneration (Bermudez-Lekerika et al., 2025). For that reason, TRPV4 is better treated as a regulatory node whose output shifts with loading, osmotic pressure, and inflammatory signaling than as a purely pathogenic or purely protective factor.

### 4.5 Mutual amplification of inflammatory responses and mechanical sensitization

In lumbar degeneration, the problem is not abnormal force alone. It is the way inflammation changes how cells read that force. Organ culture of isolated discs shows that dynamic compression combined with torsion and superimposed IL-1β leads to reduced disc height, decreased water content, downregulation of ACAN, and increased MMP-13, indicating that mechanical overload is more likely to shift toward catabolism when inflammation is present (Crump et al., 2026). Graded hydrostatic pressure models tell a similar story: low-load dynamic pressure supports survival of nucleus pulposus cells and inner annulus fibrosus cells and helps preserve ECM homeostasis, whereas high-amplitude pressure disrupts this balance through Hippo-YAP/TAZ-related apoptosis (Wang et al., 2021b). Periodic hydrostatic pressure that simulates circadian spinal motion also upregulates anabolic markers such as aggrecan, HAS2, and COL2A1 and suppresses MMP13 and COL1A1, which suggests that physiological rhythmic loading itself has anti-catabolic properties (Vieira et al., 2021). The same threshold effect appears in annulus fibrosus cells: 5% cyclic stretch maintains YAP activity and limits NF-κB nuclear translocation, whereas 12% stretch shifts the response toward pro-inflammatory stimulation (Wang et al., 2023b). Under IL-1β pre-stimulation, moderate stretch can even reverse increases in Cox2, Tnfa, Il1b, and Il6 together with downregulation of the Cav1/integrin β1/NF-κB axis (Zhang et al., 2021). Inflammation does not erase mechanoregulation; it makes tissues more sensitive to loading pattern.

Inflammation also amplifies mechanical sensitization by reshaping the receptor landscape. Chondrocyte studies show that IL-1α upregulates Piezo1 and increases basal Ca²^+^ influx, making cells more vulnerable to mechanical injury even at rest (Lee et al., 2021). In disc cells, IL-1β or TNF-α reinduces TRPA1 expression, downregulates TRPV1, and simultaneously reshapes the expression profiles of TRPV2, TRPV4, and TRPC6, which suggests that inflammation does not simply boost existing channel activity; it changes the composition of the mechanoreceptor system itself (Kameda et al., 2019). Additional evidence from osteoarticular chondrocytes indicates that either TRPA1 deletion or pharmacological antagonism downregulates IL-6, LIF, and IL-11, implicating TRPA1 in maintenance of inflammation (Nummenmaa et al., 2020). Oxidative stress adds another amplification layer: the lipid peroxidation product 4-hydroxynonenal can directly activate TRPA1 and trigger pain-related neurogenic inflammatory output (Trevisani et al., 2007). In clinical specimens, disc TBARS levels rise significantly with higher Pfirrmann grades, supporting the presence of a pathological microenvironment marked by sustained oxidative stress and TRPA1 signaling (Morales et al., 2024). Inflammation, abnormal loading, and oxidative stress therefore reinforce one another and jointly lower the activation threshold of mechanosensitive channels, pushing what would otherwise be physiological loading toward pathological signaling.

## 5 Piezo/TRP-based mechanotransduction mechanisms relevant to manual therapy

**Figure 2:**
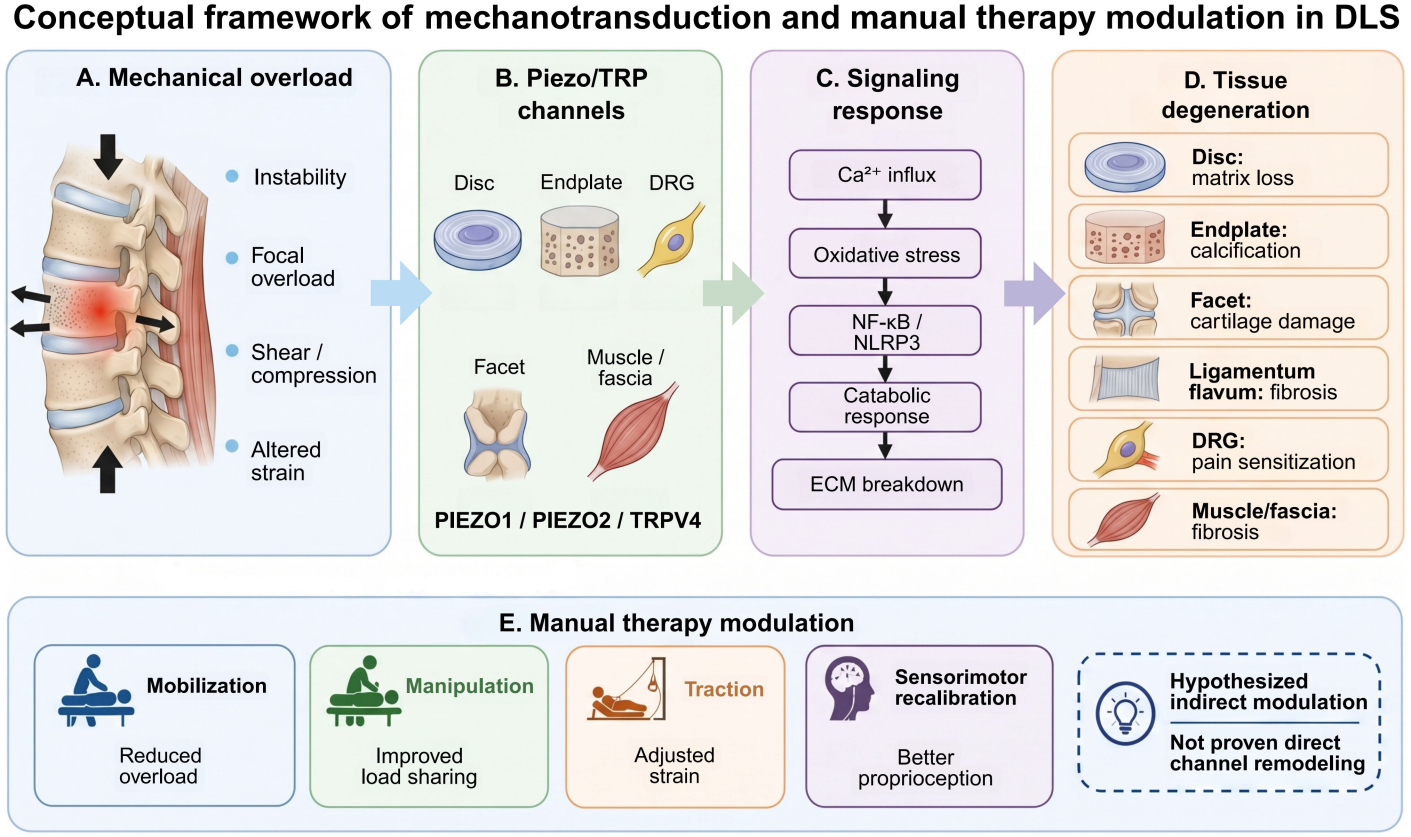
Conceptual framework of abnormal mechanotransduction and indirect modulation by manual therapy in DLS. **(A)** Degenerative lumbar spondylolisthesis (DLS) and related lumbar degeneration may lead to segmental instability, focal overload, increased shear/compression, and altered tissue strain. **(B)** Piezo1, Piezo2, and TRPV4 in the intervertebral disc, cartilage endplate, dorsal root ganglion, facet joint, and muscle/fascia system may serve as candidate readout nodes for changes in the mechanical environment. **(C)** Abnormal mechanical input may promote degeneration-related outputs through Ca²^+^ influx, oxidative stress, NF-κB/NLRP3 activation, catabolic responses, and ECM disruption. **(D)** These responses manifest across tissues as intervertebral disc matrix loss, endplate calcification, facet cartilage damage, ligamentum flavum fibrosis, dorsal root ganglion pain sensitization, and muscle/fascia fibrosis. **(E)** Manual therapy, mobilization, traction, and sensorimotor recalibration may indirectly alter the activation context of mechanosensitive channels by reducing local overload, improving load sharing, adjusting tissue strain, and enhancing proprioception. This figure proposes a testable mechanistic framework in which manual therapy may indirectly influence mechanosensitive channel-related biological processes by optimizing the local mechanical environment, whereas channel-level effects remain to be validated. Created in BioRender. HA, Y. (2026) https://BioRender.com/2c26fmv.

The biomechanical and mechanobiological evidence reviewed above gives a practical frame for thinking about manual therapy-related mechanotransduction. Current studies suggest that the mechanical stimuli delivered by manual therapy may change the conditions under which mechanosensitive ion channels such as Piezo/TRP are activated by altering segmental stress distribution and local tissue strain in the spine (Vadala et al., 2026). The effects of manual therapy may therefore extend beyond immediate anatomical adjustment. They may also involve reorganization of abnormal loading and the local mechanical microenvironment, with measurable consequences at the level of cellular mechanotransduction. That inference still rests mainly on cross-level integration of clinical, biomechanical, and basic evidence, and direct experimental support remains limited. On the basis of current cross-level evidence, manual therapy is more likely to influence Piezo/TRP-related mechanotransduction indirectly, by redistributing segmental loads and adjusting the local mechanical environment. The corresponding working model, together with potential levels of action associated with different manual therapy inputs, is illustrated in **Figures 2A–E**.

### 5.1 Clinical classification and foundational efficacy evaluation

If classification is meant to reflect mechanism, naming techniques is not enough. The more informative approach is to classify manual procedures by measurable characteristics of mechanical exposure. A systematic review of high-velocity low-amplitude procedures shows that different spinal manipulations differ fairly consistently in peak force, loading rate, and tissue force distribution, which makes it hard to treat “manual therapy” as a single homogeneous mechanical stimulus (Langenfeld et al., 2025). Three-dimensional kinematic studies of seated lumbar rotational manipulation likewise show that force, velocity, and direction can be quantified objectively (Han et al., 2025). The better units of classification are therefore the temporal profile, directional profile, and segmental target of the applied force rather than the conventional labels attached to techniques. To avoid treating different manual therapy techniques as a single mechanical exposure, this review summarizes common manual techniques, soft-tissue interventions, and experimental loading models by predominant mechanical input, level of action, and boundary of mechanistic inference (**Table 3**).

**Table 3:** Mechanical inputs and evidence boundaries across manual therapy approaches and experimental loading models.

Intervention or model	Primary mechanical input	Biological level	Mechanistic implication	References
HVLA spinal manipulation	Preload, brief peak force, low amplitude impulse	Facet joint, joint capsule, paraspinal tissue, sensory afferents	May alter segmental mobility, tissue stiffness and pain modulation	(Jun et al., 2020; Langenfeld et al., 2025).
Mobilization or rotational mobilization	Low velocity repetitive translation or rotation	Joint capsule, periarticular soft tissue, motion segment	May influence tissue viscoelasticity, fluid movement and mechanoreceptor input	(Wang et al., 2018; Savage et al., 2025).
Traction or flexion distraction	Axial distraction, decompression, foraminal and pressure changes	Intervertebral disc, endplate, nerve root, DRG	May modify compressive stress, fluid environment and osmotic loading	(Liu et al., 2021; Bernhard et al., 2025).
Soft tissue or fascial manual therapy	Shear, compression, stretch and tissue gliding	Thoracolumbar fascia, paraspinal muscle, ECM, peripheral nerve endings	May affect fascial gliding, tissue stiffness and mechanical pain sensitivity	(Javaid et al., 2025).
Manual therapy combined with exercise rehabilitation	External mechanical input with active contraction and repeated physiological loading	Muscle, fascia and sensorimotor control system	May improve load distribution, deep spinal stability and sensorimotor control	(Owen et al., 2019; George et al., 2021).
Calibrated loading or experimental model	Controlled compression, stretch, cyclic loading and stiffness modulation	Cells, organ culture, animal tissue or ex vivo tissue	Can define mechanical dose, tissue thresholds and testable mechanistic hypotheses	(Kanda et al., 2020; Salzer et al., 2023).

HVLA, high velocity low amplitude; DRG, dorsal root ganglion; ECM, extracellular matrix; DLS, degenerative lumbar spondylolisthesis.

“Mechanistic interpretation supported by current evidence” indicates plausible mechanobiological relevance. It does not indicate that manual therapy has been proven to directly regulate Piezo or TRPV channels.

On that basis, the available evidence can be grouped into four broad categories. The first is rapid thrust-dominant procedures. These studies are useful because they define instantaneous thrust and loading rate more clearly and can record changes in disc pressure *in vivo*, making them well suited for characterizing “short-duration, high-gradient mechanical input” (Lisi et al., 2006). Load distribution analyses of posterior-to-anterior standardized thrusts also show that external forces are not transmitted evenly across tissues, but instead create tissue-selective internal loading patterns (Funabashi et al., 2022). The second category includes mobilization or rotational procedures. Simulation studies indicate that these inputs differ from rapid thrusts in their disc pressure curves (Wang et al., 2018). Finite-element analyses in different body positions further show that similarly named rotational procedures do not produce identical stress focal points within tissues; what matters is less the action label than the way the load path is redistributed (Li et al., 2017). The third category includes distraction or flexion-distraction inputs. Ultrasound studies show that these procedures can generate measurable segmental separation and displacement changes and are mainly used to analyze decompression windows and stress unloading (Kruse et al., 2025). The fourth category comprises non-manual mechanical models. Ex vivo loading devices can simulate the segmental mechanical environment under defined boundary conditions with participation of trunk musculature (Matziolis et al., 2025), while dynamic pressure stimulation models show that specific pressure configurations can upregulate collagen II and aggrecan expression in nucleus pulposus cells and help define the range between homeostatic maintenance and promotion of degeneration (Tseng et al., 2021).

Clinically, the efficacy signal is present but modest. The latest Cochrane review still supports an average benefit of spinal manipulative therapy for chronic low back pain, although effect sizes are limited and heterogeneity remains substantial (de Zoete et al., 2026). Recent network meta-analyses likewise show that differences among SMT delivery modes in pain and functional outcomes are usually small, which makes dose stratification difficult if one relies on technique name alone (Nim et al., 2025). A systematic review and meta-regression reached a similar conclusion: exercise therapy and manual therapy showed no clear overall superiority when used as stand-alone interventions, although exercise therapy held a slight advantage for long-term disability outcomes, and some of the heterogeneity could be explained by sex distribution, age, and treatment duration (González‐Gómez et al., 2025). Another meta-analysis found that, in chronic non-specific low back pain, adding manual therapy to exercise did not yield a clear extra short-term analgesic effect but might be somewhat more favorable for short- and long-term disability outcomes (dos Santos et al., 2026). In older adults with chronic low back pain, an individual participant data meta-analysis suggested that SMT and guideline-recommended interventions have broadly similar effects on pain and function, making SMT best viewed as an optional component of conservative care (Jenks et al., 2022). A Medicare-based cohort study further found lower rates of escalation of care among older adults whose long-term treatment began with spinal manipulation than among those whose care began with opioid analgesics (Whedon et al., 2021). What remains unclear is who benefits most. Even after incorporating 172 candidate baseline factors, a systematic review concluded that demographic characteristics, questionnaire measures, and physical examination findings still cannot reliably predict response to manual therapy (Barbier et al., 2025). A three-arm randomized trial also showed that pre-treatment verbal suggestions can alter short-term pain relief and pressure pain threshold responses after lumbar thrust manipulation, which means the clinical “dose” is shaped by both mechanical input and context (Zaworski et al., 2025).

### 5.2 Macroscopic biomechanical effects of manual interventions

At the macroscopic level, manual interventions can produce measurable biomechanical changes. Early feasibility studies showed that transient changes in intervertebral displacement and intradiscal pressure can be recorded during lumbar manual therapy, confirming that mechanical input from manual procedures can reach the internal loading system of the motion segment (Maigne and Guillon, 2000). Simulation studies further show that spinal manipulation and mobilization generate different disc pressure curves in both amplitude and temporal configuration and therefore should not be treated as equivalent “mechanical doses” (Wang et al., 2018). Load distribution analyses of posterior-to-anterior standardized thrusts also suggest that external forces are borne mainly by the disc and the anterior and posterior longitudinal ligaments, with relatively less load on the facet joints and ligamentum flavum; manual therapy therefore seems more likely to reroute loading through selected tissues than to create a uniformly diffuse pressure field across the posterior column (Funabashi et al., 2022). In a prolonged sitting model, a single lumbar manipulation did not change static spinal or pelvic posture but did reduce trunk muscle activity and increase lumbar angular transition, which points to early changes in load sharing rather than gross postural correction (De Carvalho and Callaghan, 2022). Quantitative ultrasound studies also show that both flexion range and total sagittal segmental motion at L4/5 increase immediately after intervention and remain detectable after a short period of supine rest, regardless of whether central posterior-to-anterior mobilization or rotational mobilization is used (Savage et al., 2025). A crossover randomized trial similarly found that both mobilization and manipulation increase lumbar mobility and pressure pain threshold after a single treatment, with no stable difference between techniques (Lei et al., 2025).

These effects do not stop at displacement. Manual interventions can also modify sensory input and postural control. Randomized controlled trials show that a single lumbar manipulation can increase reliance of postural control on proprioceptive input from the lumbar segment and that patients with greater baseline lumbar proprioceptive weighting are more likely to experience stronger analgesia (Nyirö et al., 2025). Immediate local desensitization, however, does not necessarily come with better postural stability; double-blind placebo-controlled trials found only local increases in pressure pain threshold, without matching changes in postural stability metrics (Freitas et al., 2024). Likewise, targeting either the “stiffest segment” or the segment with the “lowest pain threshold” did not consistently change short-term pain outcomes or biomechanical stiffness indices. Differences appeared mainly in pressure pain thresholds, while segmental stiffness itself remained inconsistent (Nim et al., 2020). Follow-up analysis of the same trial also failed to show a systematic reduction in lumbar stiffness, which suggests that the earliest response to manual therapy may be altered mechanical pain sensitivity rather than a clear mechanical softening of the segment (Nim et al., 2021).

Distraction and decompression inputs show a similar pattern. Their macroscopic effects can be tracked through imaging and surrogate tissue measures. T2 mapping studies show that continuous traction increases T2 values in the nucleus pulposus of Pfirrmann grade II-IV discs and that the size of this change correlates strongly with improvement in ODI/VAS, suggesting that certain unloading patterns may affect disc hydration in the short term (Liu et al., 2021). In a randomized controlled trial of 424 patients with radicular pain due to disc herniation, traction combined with medication outperformed medication alone at 1 month, which suggests that adjunctive decompression may have detectable clinico-mechanical benefit (Bernhard et al., 2025). Randomized ultrasound studies also found that joint mobilization can be accompanied by increased thickness of the transversus abdominis and multifidus, raising the possibility that manual therapy supports segmental control through recruitment of deep stabilizing musculature and geometric realignment (Javaid et al., 2025). Repetitive mechanical loading can also induce biological responses in the annulus fibrosus of porcine discs, offering experimental support for a link between macroscopic load redistribution and cell-level change (Matyas et al., 2021). In broad terms, the evidence points more strongly toward adjustments in segmental kinematics, load-transfer pathways, deep stabilizing systems, and sensorimotor integration than toward immediate structural correction of degenerative tissues.

### 5.3 Micromechanical transduction based on Piezo/TRP

A plausible next step is that changes in segmental kinematics and load-transfer pathways alter the mechanical field, fluid environment, and matrix state experienced by cells, and thereby alter biology as well. In DLS, where chronic instability and repeated abnormal shear are central features, tissue responses depend on both single loading events and cumulative exposure over time, as well as on the state of the local microenvironment. Mechanically active nucleus pulposus-on-a-chip models show clear dependence of human nucleus pulposus tissue on both the intensity and the duration of compressive stimulation: loading within the physiological range tends to preserve matrix homeostasis, whereas higher loading shifts tissues toward inflammatory, catabolic, and apoptotic phenotypes (Krupkova et al., 2026). Low hydrostatic pressure can maintain functional homeostasis of nucleus pulposus cells through the TRPV4/CRT/FA complex axis (Hu et al., 2025). TRPV4 activates different cytokine networks under static and dynamic compression, which means that cellular interpretation depends on load magnitude, rhythm, and exposure duration (Easson et al., 2023). Ex vivo dynamic loading models further show that sustained compression promotes loss of the nucleus pulposus notochordal-like phenotype, whereas inhibition of integrin α5β1 delays this process, suggesting that cell-matrix adhesion complexes take part in early recognition and transduction of pathological mechanical signals (Kanda et al., 2020).

Whether a given load becomes pathological also depends on the state of the local microenvironment. Human nucleus pulposus cells can maintain a matrix-homeostatic mechanical response under non-degeneration-like pH conditions, but they shift toward a catabolic interpretation in degeneration-like acidic environments (Hodson et al., 2018). The PIEZO1-primary cilium axis can participate in degenerative transduction in growth plate cartilage under compressive stress, indicating close coupling between mechanosensitive ion channels and organellar scaffolds; by contrast, the primary cilium-PTH1R mechanosensory response within the nucleus pulposus appears to protect age-related homeostasis (Zheng et al., 2018; Chen et al., 2025). At the endplate level, IFT88 suppresses TRPV4 overactivation and calcium overload, thereby attenuating overload-related endplate calcification and disc degeneration (Dong et al., 2026). Degeneration-related ECM composition can also reset the threshold for mechanical response. Fibronectin enhances contractility, YAP activation, and NF-κB signaling in nucleus pulposus cells while suppressing proteoglycan synthesis phenotypes (Naha and Driscoll, 2023). Increased stiffness likewise reprograms energy metabolism: degeneration-like stiff matrices inhibit glycolysis in nucleus pulposus cells through MRTF-A-dependent mechanotransduction (Xu et al., 2025). Closer to end-stage injury phenotypes, stiff matrices can also drive ferroptosis of nucleus pulposus cells through YAP- and N-cadherin-related mechanotransduction (Ke et al., 2023a). Region-specific bioprinted disc models further show that the effects of stiffness and oxygen tension on matrix secretion differ between the nucleus pulposus and annulus fibrosus, underscoring the strong tissue-compartment dependence of this threshold (Kibble et al., 2025).

Current evidence still does not justify calling manual therapy a direct regulator of Piezo or TRPV4. A narrower and more defensible reading is that it may transiently modify the combined mechanical context of compression, distraction, shear, and fluid exchange, thereby altering the activation environment of mechanosensitive pathways and lowering the likelihood that abnormal mechanotransduction will be triggered. Findings from the sensory system support that layered interpretation: single-channel studies of the DRG show that mechanosensitive ion channels can continue to amplify abnormal structural inputs in the periphery (Murthy, 2023). Mechanical overload can trigger Piezo1-mediated calcium influx and GPX4-associated ferroptosis and can also drive growth plate chondrocyte apoptosis through Piezo1 (Wang et al., 2022; Chen et al., 2023). Related reviews likewise frame the potential value of Piezo-related intervention mainly as correction of abnormal mechanical environments rather than simple channel blockade (Savadipour et al., 2023). Short-term dynamic unloading, moreover, provides only partial relief in already induced disc degeneration, indicating that once pathological programs are established, reduction of load alone is not enough to reverse later cascades (Soubrier et al., 2025).

The evidence closest to manual therapy itself still comes largely from animal and surrogate models. Standardized tuina preserves disc morphology and reduces nucleus pulposus cell apoptosis more effectively than sham treatment (Ye et al., 2025). YAP/TAZ-TEAD inhibition can partially reverse mechanical stress-related ECM remodeling (Xiaojun et al., 2025), and IL-33 hydrogels targeting mechano-biological signaling transitions can also attenuate disc degeneration (Zheng et al., 2026). The NF-κB/periostin positive feedback loop initiated by PIEZO1 can continuously drive nucleus pulposus cell senescence (Wu et al., 2022), while abnormal redistribution of load alone is sufficient to promote fibrotic remodeling of the disc (Sao and Risbud, 2025). Under hydrostatic pressure, augmented chondroitin sulfate proteoglycans can shift tissue responses back toward an anabolic direction (Takeoka et al., 2021). Read together, these findings suggest that the micro-level significance of manual therapy lies less in direct channel targeting than in modulation of the spatiotemporal configuration of loading and the local microenvironment, with downstream effects on Piezo/TRP-related mechanotransduction as well as inflammatory, apoptotic, and fibrotic responses.

## 6 Evidence boundaries, stratified intervention model, and translational validation

### 6.1 Evidence boundaries

Current evidence is insufficient to show that manual therapy directly regulates abnormal mechanosensitive ion channel activity. Its mechanisms should therefore be positioned as cross-level mechanistic inferences. Trials of manual therapy for low back pain often underreport contact site, force direction, loading rate, holding time, and co-interventions. This makes it difficult to translate clinical outcomes into a reproducible “mechanical dose” (Ruzich et al., 2024). More importantly, high-level randomized evidence is drawn mainly from broad acute and subacute low back pain cohorts rather than DLS-specific populations. Even when functional improvement is observed, the contribution of the manual impulse itself remains difficult to separate from biopsychosocial support components (Bronfort et al., 2026). Thus, the evidence remains largely macroscopic, including changes in segmental stiffness, joint space, or local positional relationships. It cannot establish that symptom relief is accompanied by specific changes in the gating state of Piezo1 or TRPV4 (Jun et al., 2020).

Outcome interpretation is further constrained by the weak linear relationship between structural abnormalities and symptom burden. Lumbar degenerative parameters on MRI correlate only partially with pain and disability. Morphological grading alone is therefore insufficient as a surrogate endpoint for microscopic mechanotransduction abnormalities (Bassani et al., 2024). Low back pain dominated by nociplastic mechanisms may also involve focal neuroinflammatory activation in the sensorimotor cortex and sensorimotor dysfunction. Persistent symptoms are therefore strongly shaped by central sensitization states (Shraim et al., 2024). Sensory nerves also contribute directly to intervertebral disc ECM homeostasis, rather than serving only as passive nociceptive afferent pathways (Hu et al., 2022). Sustained transcriptomic remodeling of the dorsal root ganglion in chronic discogenic pain models further suggests that tissue repair and symptom resolution do not necessarily occur in parallel (Caparaso et al., 2026).

For these reasons, evaluation of manual therapy is shifting from overall efficacy assessment toward cross-level bridging endpoints. Combined assessment of MRI features and inflammatory biomarkers may identify pain-related degenerative phenotypes more effectively than morphology alone (Chen et al., 2024b). Longitudinal cohort findings further suggest that ECM-, inflammation-, and pain-sensitization-related biomarkers do not predict structural progression and pain evolution in the same way. Indicators at different levels may therefore correspond to different disease processes, and a single imaging label is unlikely to capture patient heterogeneity (Goode et al., 2023). Single-cell atlases have identified an advanced fibrotic-like nucleus pulposus cell subpopulation marked by upregulated serglycin. This provides a traceable molecular readout for testing whether an intervention truly alters degenerative cell states (Chen et al., 2024a). Based on current evidence, classification strategies based on pain mechanisms and the local microenvironment may be more useful for evaluating whether standardized mechanical inputs produce consistent imaging, molecular, physiological, and symptom trajectories (Nijs et al., 2024). Until such evidence is established, clinical studies cannot readily translate treatment frequency into a microscopic mechanobiological dose. They also cannot map specific manual procedures onto defined mechanosensitive channel responses (Anderson et al., 2024).

Overall, the effects of manual therapy in DLS are mainly reflected in modulation of segmental motion, load transmission, myofascial tension, and sensorimotor control. Because a continuous evidence chain linking mechanical input, tissue response, and symptom improvement is still lacking, future studies should establish a stratified intervention model according to disease stage, symptom source, and tissue reversibility.

### 6.2 Stratified intervention model

On the basis of these evidence boundaries, manual therapy cannot yet be equated with clinical regulation of mechanosensitive ion channels. Clinical translation should instead return to modulation of the mechanical environment in DLS. Stratification should consider disease course, pain source, functional status, and tissue reversibility. Natural history studies of DLS suggest that slip progression, segmental stability, imaging degeneration, and symptom burden do not necessarily progress synchronously. Treatment escalation based only on imaging severity may therefore overlook patient heterogeneity (Atalay et al., 2023). The KNGF guideline organizes management of low back pain and lumbosacral radicular syndrome according to the risk of persistent symptoms. This indicates that treatment intensity should match pain risk and functional limitation (Apeldoorn et al., 2024). Low back pain may also involve nociceptive, neuropathic, and nociplastic mechanisms simultaneously. Literature on individualized pain management has incorporated trigger points, neuromuscular imaging, and rehabilitation decision-making into the same evaluative framework (Bubnov, 2012; Knezevic et al., 2021). The stratified model proposed here emphasizes integrated interpretation of structural imaging, functional assessment, and clinical symptoms. It is not based solely on technical invasiveness or the sequence of treatment escalation.

In the early or function-dominant stage, the main problems include reversible segmental hypomobility, abnormal postural loading, insufficient paraspinal muscle recruitment, impaired thoracolumbar fascial sliding, and sensorimotor control imbalance. A recent multinational randomized controlled trial in DLS showed that integrated nonsurgical care provided additional relief of back and leg pain compared with usual care, without treatment-related serious adverse events (Kim et al., 2026). Low back pain guidelines include exercise, manual therapy, and patient education as components of non-pharmacological management. Exercise modalities can also be selected according to functional deficits (Owen et al., 2019; George et al., 2021). At this level, manual therapy is best understood as a way to modulate mechanical input and local load distribution, rather than to reverse structural degeneration. It may act together with postural management, motor-control training, and core-stability training to influence segmental kinematics, deep stabilizing muscle recruitment, fascial sliding, and sensory feedback. Reviews of degenerative spinal kinematics show altered segmental and coupled motion under degenerative conditions, providing a biomechanical basis for early functional modulation (Widmer et al., 2019).

Beyond structural imaging, functional ultrasound assessment may provide additional stratification information. MRI and CT can reveal structural degeneration, but they have limited ability to capture real-time contraction, sliding, stiffness, and shear strain in the paraspinal muscles, transversus abdominis, and thoracolumbar fascia. Ultrasound can help fill this gap and may bridge structural imaging with muscle-fascia functional behavior (Cheung et al., 2020). Reviews of thoracolumbar fascia ultrasound and studies using shear-wave elastography suggest that local fascial and paraspinal muscle mechanical states can be incorporated into functional diagnosis (Liu et al., 2024a; Pirri et al., 2024). These indicators cannot replace direct assessment of Piezo/TRP channel activity. They can, however, indicate whether local tissues are characterized by high tension, high stiffness, reduced sliding, or abnormal strain. In intervertebral disc studies, TRP channel expression varies with degeneration and back pain status. This supports functional imaging as an indirect observational window linking local mechanical states with the mechanosensitive channel hypothesis (Sadowska et al., 2019).

The intermediate stage mainly addresses persistent myofascial pain sources. If reproducible local pain, paraspinal stiffness, impaired thoracolumbar fascial sliding, or trigger points persist after postural management, manual therapy, and motor-control training, ultrasound-guided targeted intervention may be considered. Early exploratory fascial ultrasound studies have extended potential targets from muscular trigger points to abnormal fascial regions. Reduced mobility, adhesion, tissue heterogeneity, or altered local blood flow may contribute to myofascial pain and postural imbalance. However, these sonographic signs and their therapeutic relevance still require validation in controlled studies (Bubnov and Kalika, 2021). Randomized trials combining dry needling with shear-wave elastography suggest that resting stiffness in some lumbar muscles may change in the short term. Reviews of myofascial pain in low back pain nevertheless consider long-term evidence insufficient (Koppenhaver et al., 2021; Dach and Ferreira, 2023). The advantages of ultrasound-guided trigger point intervention mainly relate to localization, safety, and procedural reproducibility (Diep et al., 2020). Clinical comparative studies suggest that visualization may assist trigger point identification, needle-tip observation, and short-term pain evaluation (Bubnov and Wang, 2013). Ultrasound-guided dry needling or other local targeted procedures may therefore serve as a supplementary pathway between conservative treatment and further interventional management.

The late or refractory stage is the point at which structural or neuromodulatory escalation should be considered. At this stage, symptoms should be consistent with spinal canal stenosis, nerve root compression, discogenic pain, or vertebrogenic pain. Function-oriented treatment and soft-tissue targeted management should also be insufficient for symptom control. Reviews of lumbar spinal stenosis still recommend initial nonsurgical management and reserve surgical evaluation for selected patients with persistent pain and activity limitation (Katz et al., 2022). Guidelines on interventions for chronic spinal pain provide cautious recommendations for several commonly used procedures. Disc-related treatments, radiofrequency procedures, neuromodulation, or surgical evaluation are therefore appropriate only under strict indications (Busse et al., 2025). Piezo1 links abnormal mechanical loading, inflammatory responses, and cell-fate imbalance in intervertebral disc degeneration. This mechanistic background supports separating late-stage structural pain sources from early and intermediate functional abnormalities (Guo et al., 2022).

Overall, stratified intervention helps place manual therapy within distinct disease stages and mechanical contexts of DLS. Load states, tissue reversibility, and pain sources differ across stages. The effects of manual therapy on local strain, load transmission, and sensory feedback should therefore also be discussed in a stratified manner. On this basis, future studies should validate the translational significance of the Piezo/TRP-related mechanotransduction hypothesis in DLS by integrating mechanical dose, imaging phenotypes, biomarkers, and clinical trajectories.

### 6.3 Future directions for translational validation

The stratified model described above preliminarily defines the position of manual therapy in conservative DLS management. Whether different strata correspond to specific tissue mechanical responses and clinical outcomes still requires validation. The effects of manual therapy on the mechanical environment, mechanotransduction, tissue responses, and symptoms should therefore be treated as a working model. A continuous causal relationship across these levels remains to be confirmed. Within this model, manual therapy is understood mainly as a means of modulating local mechanical input and load-transmission states. Piezo1, Piezo2, and TRPV4 may participate in tissue sensing and responses to those mechanical changes.

**Figure 3:**
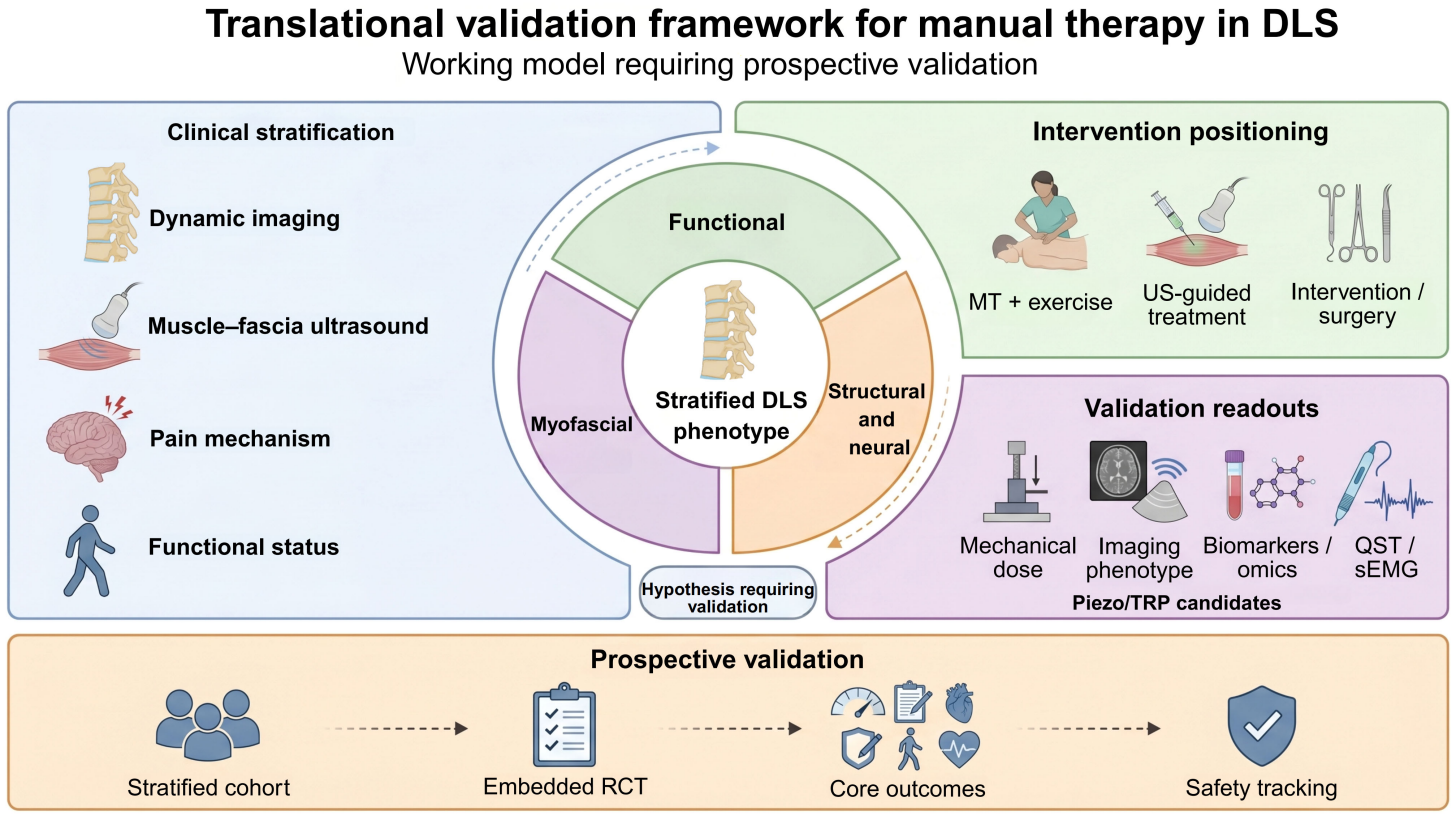
Prospective validation framework for the manual therapy-related mechanotransduction hypothesis in DLS. The left panel shows that clinical stratification should integrate dynamic imaging, muscle-fascia ultrasound, pain mechanisms, and functional status to identify function-dominant, myofascial-dominant, and structural/neural-dominant DLS phenotypes. The upper-right panel shows phenotype-specific intervention positioning, including manual therapy plus exercise, ultrasound-guided treatment, and interventional or surgical escalation. The lower-right panel shows validation readouts, including mechanical dose, imaging phenotype, biomarkers/omics, QST/sEMG, and Piezo/TRP candidate indicators. The bottom band proposes a prospective validation pathway, including stratified cohorts, embedded randomized controlled trials, core outcome assessment, and safety tracking, to provide a structured research framework for the manual therapy-related mechanotransduction hypothesis. Created in BioRender. HA, Y. (2026) https://BioRender.com/6vxsgc0.

Current evidence is concentrated at two relatively clear levels. First, mechanical stimulation can affect human intervertebral disc cells and matrix-related responses, although loading modes, cell sources, and degeneration status remain heterogeneous (Ruffilli et al., 2023). Second, the procedural types and reporting methods grouped under “manual therapy” vary substantially. Future studies should therefore not equate the treatment label with a single mechanical exposure (Wenger et al., 2023). Whether manual inputs in patients with DLS alter Piezo/TRP-related mechanotransduction, and whether such changes contribute to tissue remodeling and symptom improvement, remains an intermediate link requiring validation. To avoid presenting this inference as an established mechanism, this review integrates clinical stratification, intervention positioning, and validation readouts into a prospective validation framework (**Figure 3**).

The first step in validating this working model is to translate manual therapy into a recordable and comparable mechanical dose. Future DLS studies should report at least the contact site, target segment, loading direction, peak force, loading rate, holding time, treatment frequency, and co-interventions. This would convert the broad description of “receiving manual therapy” into an analyzable mechanical exposure. Actual mechanical exposure is also influenced by degenerative status, tissue tolerance, and practitioner judgment. Because DLS populations are mainly composed of middle-aged and older adults, age-related contextual factors may alter peak force, peak acceleration, and peak velocity. Objective recording of mechanical dose is therefore needed in future studies (Maiers et al., 2025). Once mechanical dose is recorded, studies should examine whether it corresponds to short-term biological changes. Exploratory studies have found that spinal manipulation at different force levels can affect blood oxidative stress markers and inflammation-resolving mediators in healthy participants. Although these findings cannot be directly extrapolated to DLS, they suggest that standardized mechanical dose can be paired with peripheral biological indicators (Duarte et al., 2025).

Beyond mechanical dose and peripheral indicators, the mechanobiological phenotype of DLS requires reliable structural and functional imaging definitions. At the basic level, standing and flexion-extension radiographs, conventional MRI, and necessary dynamic assessments can record slip magnitude, segmental mobility, intervertebral disc degeneration, facet joint changes, ligamentum flavum thickness, spinal canal stenosis, and paraspinal muscle status. Imaging thresholds and measurement methods for sagittal segmental instability still require standardization (Elmose et al., 2022). At the functional and tissue-mechanics levels, T2 mapping, magnetic resonance elastography, ultrasound shear-wave elastography, and assessment of thoracolumbar fascial sliding may provide additional information on tissue hydration, stiffness, and myofascial motion (Bubnov, 2013; Bubnov et al., 2018). On this basis, radiomics can integrate muscle morphology, texture features, and clinical variables to refine patient stratification and prognostic assessment. In patients with DLS, MRI radiomics of the paraspinal muscles combined with clinical variables has been used to predict postoperative outcomes. This approach may provide additional support for prognostic stratification (Yu et al., 2025).

After imaging phenotypes have been defined, molecular, omics, and sensory functional indicators can serve as bridging endpoints. These endpoints can track associations among macroscopic mechanical input, tissue response, and neural functional change. Blood inflammatory factors, ECM metabolic markers, and neurogenic inflammation indicators can describe the inflammatory and matrix-remodeling background. Large observational cohorts of chronic low back pain have shown that circulating factors such as IL-6, IL-1ra, TNF, and leptin differ across levels of pain and disability (Enrico et al., 2025). Piezo/TRP channel expression, Ca²^+^ influx, mechanically activated currents, and downstream pathways such as YAP/TAZ, NF-κB, and NLRP3 can be validated using intraoperative tissues, ex vivo loading systems, animal models, or organ-on-chip platforms. Recent reviews of Piezo1 and intervertebral disc degeneration also position these indicators mainly as experimental validation readouts for mechanical stress-related degenerative mechanisms (Li et al., 2026). RNA-seq, single-cell transcriptomics, and spatial transcriptomics may support mechanism exploration and hypothesis generation (Wang et al., 2023a). Their value as clinical stratification indicators, however, requires further validation (Chen et al., 2024c). For sensory and motor-control readouts, pressure pain threshold, temporal summation, conditioned pain modulation, and the flexion-relaxation phenomenon of the lumbar extensor muscles on surface electromyography may help assess pain sensitization, proprioception, and neuromuscular control. Existing QST and sEMG studies in low back pain remain heterogeneous in stimulus type, test site, recording method, and population definition. More standardized testing protocols are therefore needed (Gouteron et al., 2021; Goodman et al., 2025).

Once these validation indicators are clarified, future studies should incorporate them into executable clinical research designs. Prospective stratified cohorts may be established, with embedded randomized controlled trials in suitable populations. Eligible participants should preferably have imaging-confirmed Meyerding grade I–II DLS, no urgent surgical indication, and willingness to receive conservative treatment. They should be pre-stratified according to the function-dominant, myofascial pain-dominant, and structural or neural compression-dominant characteristics described above. Follow-up should synchronously record mechanical dose, imaging phenotype, ultrasound functional indicators, molecular or omics bridging endpoints, QST, electromyography, proprioceptive indicators, pain, ODI or RMDQ, quality of life, walking capacity, medication changes, treatment escalation, and adverse events. Given the long-standing inconsistency of outcome measures in manual therapy trials for low back pain, future studies should also adopt core outcome sets and unified follow-up frameworks (Brier et al., 2026).

The clinical value of mechanobiological phenotyping ultimately lies in patient selection, prognostic judgment, and treatment planning (Wirth and Schweinhardt, 2023). Future studies should integrate mechanical dose, imaging phenotype, molecular and sensory functional readouts, and patient-reported outcomes to clarify response characteristics across DLS subtypes. If longitudinal evidence is gradually established, manual therapy in conservative DLS management will no longer be limited to empirical intervention. It may instead be incorporated into a clinical decision-making framework based on mechanistic stratification.

## 7 Conclusion and perspectives

Taken together, DLS and related lumbar degenerative conditions should be understood through interactions among segmental mechanical imbalance, tissue compartment-specific responses, mechanosensitive channel abnormalities, and altered sensorimotor control. This review integrates structural instability, local mechanical microenvironmental changes, mechanosensitive ion channel responses, and manual therapy-related modulation of mechanical input into a cross-level working model. The model highlights potential links among structural degeneration, mechanosensing, and conservative intervention. Because imaging-defined slippage, segmental instability, pain burden, and responses to conservative treatment do not follow a simple linear relationship, future DLS research should incorporate stability, symptom source, and functional status into stratified analyses (Vanti et al., 2021). This framework may guide subsequent mechanistic validation and clinical stratification studies.

The evidence boundaries remain important. DLS-specific evidence is drawn mainly from epidemiology, imaging phenotypes, natural history, conservative treatment, and clinical outcomes. Studies of integrated nonsurgical care generally support improvements in pain and function, but they do not demonstrate immediate correction of the slipped structure. They also do not directly support regulation of Piezo/TRP-related pathways (Kim et al., 2026). Evidence on Piezo1, Piezo2, and TRPV4 is derived mainly from intervertebral disc degeneration, cartilage endplate studies, osteoarthritis, dorsal root ganglia, animal models, and ex vivo loading systems. The framework proposed in this review therefore remains a mechanistic inference, and the relevant clinical causal relationships require further validation.

At the mechanistic level, Piezo and TRP-related channels may link macroscopic mechanical abnormalities with cellular and neural responses. An abnormal mechanical microenvironment may induce differential channel responses across tissue compartments and jointly affect clinical manifestations through inflammation, ECM metabolism, fibrosis, pain sensitization, and altered sensorimotor control (Swahn et al., 2024; Ke et al., 2025). Manual therapy should be positioned as a means of modulating mechanical input. Its effects may relate to contact force, segmental motion, load transmission, tissue strain, and sensory feedback. Future studies need to record specific mechanical doses and avoid explaining clinical or molecular outcomes solely by treatment frequency (Nyirö et al., 2024). Clinical translation will require MRI, dynamic imaging, ultrasound assessment, and multimodal data to identify differences in structural instability, myofascial function, and pain mechanisms. After adequate validation, these measures may support patient stratification and stepwise treatment selection (Bubnov, 2010; Cina and Galbusera, 2024).

Future research should advance this framework from a mechanistic hypothesis to a testable working model. First, DLS-specific clinical cohorts and mechanistic nested studies with accessible tissue samples should be established. These studies should record slip grade, segmental stability, myofascial status, pain phenotype, and treatment trajectory. Second, the mechanical dose of manual therapy should be standardized, including at least contact site, target segment, direction, amplitude, velocity, duration, and frequency, with simultaneous recording of co-interventions. Third, MRI, dynamic imaging, ultrasound elastography, and myofascial functional assessment should be linked with Piezo1, Piezo2, and TRPV4 expression, inflammatory factors, ECM metabolism, and pain-related indicators. Subsequent mechanistic validation may be better achieved using combined models that assess cell behavior, ECM composition, and tissue mechanical function simultaneously (Salzer et al., 2023). Ultimately, future studies should determine whether standardized manual mechanical input can induce measurable tissue mechanical or neural responses in specific DLS subtypes. They should also test whether these responses are associated with symptom improvement, functional recovery, and risk of treatment escalation. Only after a longitudinal evidence chain links manual mechanical exposure, tissue response, and clinical outcome can the mechanotransduction framework support stratified intervention and efficacy prediction in DLS.

## References

[B1] Alonso F. Kirkpatrick C. M. Jeong W. Fisahn C. Usman S. Rustagi T. . (2017). Lumbar facet tropism: a comprehensive review. World Neurosurg. 102, 91–96. doi: 10.1016/j.wneu.2017.02.114 28279769

[B2] Anderson B. R. Whedon J. M. Herman P. M. (2024). Dosing of lumbar spinal manipulative therapy and its association with escalated spine care: a cohort study of insurance claims. PloS One 19, e0283252. doi: 10.1371/journal.pone.0283252 38181030 PMC10769084

[B3] Apeldoorn A. T. Swart N. M. Conijn D. Meerhoff G. A. Ostelo R. W. (2024). Management of low back pain and lumbosacral radicular syndrome: the guideline of the Royal Dutch Society for Physical Therapy (KNGF). Eur. J. Phys. Rehabil. Med. 60, 292–318. doi: 10.23736/s1973-9087.24.08352-7 38407016 PMC11112513

[B4] Assaraf E. Blecher R. Heinemann-Yerushalmi L. Krief S. Carmel Vinestock R. Biton I. E. . (2020). Piezo2 expressed in proprioceptive neurons is essential for skeletal integrity. Nat. Commun. 11, 3168. doi: 10.1038/s41467-020-16971-6 32576830 PMC7311488

[B5] Atalay B. Gadjradj P. S. Sommer F. S. Wright D. Rawanduzy C. Ghogawala Z. . (2023). Natural history of degenerative spondylolisthesis: a systematic review and meta-analysis. World Neurosurg. 176, e634–e643. doi: 10.1016/j.wneu.2023.05.112 37271258

[B6] Barbier G. Picchiottino M. Delafontaine A. Goncalves G. Bussières A. Cottin F. . (2025). Baseline individual factors associated with clinical outcomes in adults with non-specific low back pain following manual therapy: a systematic review. BMC Complementary Med. Ther. 25, 330. doi: 10.1186/s12906-025-04975-y 40968376 PMC12447601

[B7] Bassani T. Colombini A. Pallotta L. Sconfienza L. M. Albano D. Brayda-Bruno M. (2024). Association between MRI measurements of lumbar spine alterations and self-reported outcomes of pain and disability in subjects with non-specific low back pain. Eur. Spine J. Off. Publ. Eur. Spine Soc Eur. Spinal Deform. Soc Eur. Sect. Cerv. Spine Res. Soc 33, 4572–4580. doi: 10.1007/s00586-024-08449-6 39164509

[B8] Bermudez-Lekerika P. Tseranidou S. Kanelis E. Nüesch A. Crump K. B. Alexopoulos L. G. . (2025). Ex vivo and *in vitro* proteomic approach to elucidate the relevance of IL-4 and IL-10 in intervertebral disc pathophysiology. JOR Spine 8, e70048. doi: 10.1002/jsp2.70048 39931581 PMC11808320

[B9] Bernhard E. Hittinger-Roux A. Delaplace H. Pauvele L. Charlot I. Geoffroy M. . (2025). Lumbar tractions in radicular pain caused by herniated disc: randomised, open-label, superiority, and controlled trial on 424 participants. J. Clin. Med. 14, 5192. doi: 10.3390/jcm14155192 40806815 PMC12347135

[B10] Bialosky J. E. Beneciuk J. M. Bishop M. D. Coronado R. A. Penza C. W. Simon C. B. . (2018). Unraveling the mechanisms of manual therapy: modeling an approach. J. Orthopaedic Sports Phys. Ther. 48, 8–18. doi: 10.2519/jospt.2018.7476 29034802

[B11] Bojairami I. E. Driscoll M. (2021). Coordination between trunk muscles, thoracolumbar fascia, and intra-abdominal pressure toward static spine stability. Spine 47, E423–E431. doi: 10.1097/brs.0000000000004223 34545044

[B12] Brandl A. Egner C. Schleip R. (2021). Immediate effects of myofascial release on the thoracolumbar fascia and osteopathic treatment for acute low back pain on spine shape parameters: a randomized, placebo-controlled trial. Life. 11, 845. doi: 10.3390/life11080845 34440589 PMC8399614

[B13] Brandl A. Wilke J. Egner C. Reer R. Schmidt T. Schleip R. (2023). Thoracolumbar fascia deformation during deadlifting and trunk extension in individuals with and without back pain. Front. Med. 10. doi: 10.3389/fmed.2023.1177146 37342497 PMC10278943

[B14] Brier M. Phalip J. Delorme B. Schwendenmann Y. Salmon M. Bicout D. J. . (2026). Assessing the impact of manual therapy in the management of nonspecific low back pain: a scoping review of the outcomes used in clinical trials. BMC Complementary Med. Ther. 26, 111. doi: 10.1186/s12906-026-05288-4 41668102 PMC13014839

[B15] Bronfort G. Meier E. N. Leininger B. Schneider M. Evans R. Greco C. . (2026). Spinal manipulation and clinician-supported biopsychosocial self-management for acute back pain. JAMA 335, 497. doi: 10.1001/jama.2025.21990 41460638 PMC12750334

[B16] Bubnov R. (2010). The use of trigger point “dry” needling under ultrasound guidance for the treatment of myofascial pain (technological innovation and literature review). Lik. Sprava (5–6), 56–64. 21485754

[B17] Bubnov R. V. (2012). Evidence-based pain management: is the concept of integrative medicine applicable? EPMA J. 3, 13. doi: 10.1186/1878-5085-3-13 23088743 PMC3533862

[B18] Bubnov R. (2013). Ultrasound guided injections of platelets rich plasma for muscle injury in professional athletes. Comparative study. Med. Ultrason. 15, 101–105. doi: 10.11152/mu.2013.2066.152.rb1vy2 23702498

[B19] Bubnov R. V. Wang J. (2013). Clinical comparative study for ultrasound-guided trigger-point needling for myofascial pain. Med. Acupunct. 25, 437–443. doi: 10.1089/acu.2013.0973 36698420

[B20] Bubnov R. V. Kalika L. Babenko L. (2018). AB1199 dynamic ultrasound for multilevel evaluation of motion and posture in lower extremity and spine. Ann. Rheumatol. Dis. 77, 1699. doi: 10.1136/annrheumdis-2018-eular.3949

[B21] Bubnov R. Kalika L. (2021). POS1284 fascial ultrasound: the context for dry needling trigger points in treatment of myofascial pain, postural imbalance. Ann. Rheumatol. Dis. 80, 924–925. doi: 10.1136/annrheumdis-2021-eular.3843

[B22] Bubnov R. Kalika L. (2022). AB1199 the role of thoracolumbar fascia ultrasound in low back pain - implication for guided dry needling. Ann. Rheumatol. Dis. 81, 1714. doi: 10.1136/annrheumdis-2022-eular.1936

[B23] Busse J. W. Genevay S. Agarwal A. Standaert C. J. Carneiro K. Friedrich J. . (2025). Commonly used interventional procedures for non-cancer chronic spine pain: a clinical practice guideline. BMJ 388, e079970. doi: 10.1136/bmj-2024-079970 39971339

[B24] Bydon M. Alvi M. A. Goyal A. (2019). Degenerative lumbar spondylolisthesis. Neurosurg. Clin. N. Am. 30, 299–304. doi: 10.1016/j.nec.2019.02.003 31078230

[B25] Cai X. Sun M. Huang Y. Liu Z. Liu C. Du C. . (2020). Biomechanical effect of L4–L5 intervertebral disc degeneration on the lower lumbar spine: a finite element study. Orthop. Surg. 12, 917–930. doi: 10.1111/os.12703 32476282 PMC7307239

[B26] Cambria E. Arlt M. J. E. Wandel S. Krupkova O. Hitzl W. Passini F. S. . (2020). TRPV4 inhibition and CRISPR-Cas9 knockout reduce inflammation induced by hyperphysiological stretching in human annulus fibrosus cells. Cells 9, 1736. doi: 10.3390/cells9071736 32708074 PMC7407144

[B27] Cambria E. Heusser S. Scheuren A. C. Tam W. K. Karol A. A. Hitzl W. . (2021). TRPV4 mediates cell damage induced by hyperphysiological compression and regulates COX2/PGE2 in intervertebral discs. JOR Spine 4, e1149. doi: 10.1002/jsp2.1149 34611585 PMC8479521

[B28] Cao T. Peng F. Wang W. Jing X. Chen F. Sun M. . (2026). Compressive stress induces cartilage endplate degeneration through the Piezo1/YAP-TEAD/NLRP3 axis. Life Sci. 388, 124195. doi: 10.1016/j.lfs.2026.124195 41506536

[B29] Cao B. Zuo Y. Xu Y. Wu F. Du H. Hou Y. . (2023). Correlation between fat infiltration of paraspinal muscle and L4 degenerative lumbar spondylolisthesis in asymptomatic adults. Asian J. Surg. 46, 834–840. doi: 10.1016/j.asjsur.2022.08.097 36096928

[B30] Caparaso S. M. Sankaranarayanan I. Lillyman D. J. Price T. J. Wachs R. A. (2026). Single-nuclei RNA sequencing reveals distinct transcriptomic signatures of rat dorsal root ganglia in a chronic discogenic low back pain model. J. Pain 39, 105590. doi: 10.1016/j.jpain.2025.105590 41207405 PMC12767683

[B31] Chan A. K. Sharma V. Robinson L. C. Mummaneni P. V. (2019). Summary of guidelines for the treatment of lumbar spondylolisthesis. Neurosurg. Clin. N. Am. 30, 353–364. doi: 10.1016/j.nec.2019.02.009 31078236

[B32] Chen F. Chen S. Peng F. Du Y. Li J. Fan Y. . (2025). PIEZO1‐primary cilia axis mediates compressive stress‐induced growth plate degeneration and ossification in adolescent idiopathic scoliosis. JOR Spine 8, e70133. doi: 10.1002/jsp2.70133 41194970 PMC12585761

[B33] Chen F. Lei L. Chen S. Zhao Z. Huang Y. Jiang G. . (2024a). Serglycin secreted by late-stage nucleus pulposus cells is a biomarker of intervertebral disc degeneration. Nat. Commun. 15, 47. doi: 10.1038/s41467-023-44313-9 38167807 PMC10761730

[B34] Chen S. Li Z. Zhang J. Liu H. (2026). Piezo1 at the crossroads: mediating inflammation and mechanical stress in joint disorders. Joint Bone Spine 93, 105953. doi: 10.1016/j.jbspin.2025.105953 40738358

[B35] Chen F. Sun M. Peng F. Lai Y. Jiang Z. Zhang W. . (2023). Compressive stress induces spinal vertebral growth plate chondrocytes apoptosis via Piezo1. J. Orthop. Res. Off. Publ. Orthop. Res. Soc 41, 1792–1802. doi: 10.1002/jor.25527 36722421

[B36] Chen X. Wang W. Cui P. Li Y. Lu S. (2024b). Evidence of MRI image features and inflammatory biomarkers association with low back pain in patients with lumbar disc herniation. Spine J. Off. J. N. Am. Spine Soc 24, 1192–1201. doi: 10.1016/j.spinee.2024.02.006 38360179

[B37] Chen Y. Zhang L. Shi X. Han J. Chen J. Zhang X. . (2024c). Characterization of the nucleus pulposus progenitor cells via spatial transcriptomics. Adv. Sci. (Weinh) 11, 2303752. doi: 10.1002/advs.202303752 38311573 PMC11095158

[B38] Cheng Z. Wu Z. Wu M. Xie L. Chen Q. (2025). Piezo2 in mechanosensory biology: from physiological homeostasis to disease‐promoting mechanisms. Cell Proliferation 59, e70112. doi: 10.1111/cpr.70112 40781923 PMC12774628

[B39] Cheung W. K. Cheung J. P. Y. Lee W.-N. (2020). Role of ultrasound in low back pain: a review. Ultrasound Med. Biol. 46, 1344–1358. doi: 10.1016/j.ultrasmedbio.2020.02.004 32192782

[B40] Cina A. Galbusera F. (2024). Advancing spine care through AI and machine learning: overview and applications. EFORT Open Rev. 9, 422–433. doi: 10.1530/eor-24-0019 38726988 PMC11099586

[B41] Crump K. B. Muñoz-Moya E. de Graaf K. Bermudez-Lekerika P. Schwendener N. Zech W. . (2026). Complex mechanical loading and pro-inflammatory cytokines in intervertebral disc degeneration. JOR Spine 9, e70159. doi: 10.1002/jsp2.70159 41608144 PMC12835194

[B42] Dach F. Ferreira K. S. (2023). Treating myofascial pain with dry needling: a systematic review for the best evidence-based practices in low back pain. Arq. Neuropsiquiatr. 81, 1169–1178. doi: 10.1055/s-0043-1777731 38157883 PMC10756779

[B43] De Carvalho D. E. Callaghan J. P. (2022). The effect of lumbar spinal manipulation on biomechanical factors and perceived transient pain during prolonged sitting: a laboratory-controlled cross-sectional study. Chiropr. Man. Ther. 30, 62. doi: 10.1186/s12998-022-00472-y 36585725 PMC9805135

[B44] de Zoete A. Innocenti T. Petrozzi M. J. van Middelkoop M. Assendelft W. J. de Boer M. R. . (2026). Spinal manipulative therapy for adults with chronic low back pain. Cochrane Database Systematic Rev. 2026, CD008112. doi: 10.1002/14651858.cd008112.pub3 41494147 PMC12774432

[B45] Diep D. Chen K. J. Q. Kumbhare D. (2020). Ultrasound-guided interventional procedures for myofascial trigger points: a systematic review. Reg. Anesth. Pain Med. 46, 73–80. doi: 10.1136/rapm-2020-101898 33159004

[B46] Dong Z.-R. Wang J. Huang Y.-K. Ding W. He H.-X. Yuan G.-C. . (2026). Ciliary IFT88 inhibits intervertebral disc degeneration under excessive mechanical stress by regulating endplate cartilage calcification. J. Adv. Res. 81, 157–173. doi: 10.1016/j.jare.2025.05.042 40441296 PMC12957827

[B47] dos Santos E. C. S. dos Santos A. T. da Silva N. A. Carneiro S. R. Rampazo É.P. Magalhães M. O. (2026). Effectiveness of adding manual therapy to exercise for pain and disability in chronic non-specific low back pain: a systematic review and meta-analysis. Musculoskelet. Sci. Pract. 82, 103508. doi: 10.1016/j.msksp.2026.103508 41643247

[B48] Duarte F. C. K. Funabashi M. Starmer D. Partata W. A. (2025). Preliminary insights into the effects of spinal manipulation therapy of different force magnitudes on blood biomarkers of oxidative stress and pro-resolution of inflammation mediators. Chiropr. Man. Ther. 33, 8. doi: 10.1186/s12998-025-00575-2 39966844 PMC11837322

[B49] Easson G. W. D. Savadipour A. Anandarajah A. Iannucci L. E. Lake S. P. Guilak F. . (2022). Modulation of TRPV4 protects against degeneration induced by sustained loading and promotes matrix synthesis in the intervertebral disc. FASEB J. Off. Publ. Fed. Am. Soc Exp. Biol. 37, e22714. doi: 10.1096/fj.202201388r 36583692 PMC10291737

[B50] Easson G. W. D. Savadipour A. Gonzalez C. Guilak F. Tang S. Y. (2023). TRPV4 differentially controls inflammatory cytokine networks during static and dynamic compression of the intervertebral disc. JOR Spine 6, e1282. doi: 10.1002/jsp2.1282 38156056 PMC10751971

[B51] Eijkelkamp N. Linley J. E. Torres J. M. Bee L. Dickenson A. H. Gringhuis M. . (2013). A role for Piezo2 in EPAC1-dependent mechanical allodynia. Nat. Commun. 4, 1682. doi: 10.1038/ncomms2673 23575686 PMC3644070

[B52] El-Monajjed K. Driscoll M. (2021). Investigation of reaction forces in the thoracolumbar fascia during different activities: A mechanistic numerical study. Life. 11, 779. doi: 10.3390/life11080779 34440523 PMC8400736

[B53] Elmose S. F. Andersen G. O. Carreon L. Y. Sigmundsson F. G. Andersen M. O. (2022). Radiological definitions of sagittal plane segmental instability in the degenerative lumbar spine – a systematic review. Glob. Spine J. 13, 523–533. doi: 10.1177/21925682221099854 35606897 PMC9972266

[B54] Ely E. V. Lenz K. L. Paradi S. G. Ack S. Behrmann A. Dunivan S. . (2025). Chondrocyte-specific knockout of Piezo1 and Piezo2 protects against post-traumatic osteoarthritis structural damage and pain in mice. Arthritis Res. Ther. 27, 152. doi: 10.1186/s13075-025-03620-w 40684207 PMC12275339

[B55] Enrico V. T. Anderst W. Bell K. M. Coelho J. P. Darwin J. Delitto A. . (2025). Plasma pro‐ and anti‐inflammatory cytokines in an observational chronic low back pain cohort. JOR Spine 8, e70095. doi: 10.1002/jsp2.70095 40964420 PMC12439354

[B56] Fearing B. V. Hernandez P. A. Setton L. A. Chahine N. O. (2018). Mechanotransduction and cell biomechanics of the intervertebral disc. JOR Spine 1, e1026. doi: 10.1002/jsp2.1026 30569032 PMC6296470

[B57] Freitas J. P. Corrêa L. A. Bittencourt J. V. Armstrong K. M. Meziat-Filho N. Nogueira L. A. C. (2024). One spinal manipulation session reduces local pain sensitivity but does not affect postural stability in individuals with chronic low back pain: A randomised, placebo-controlled trial. Chiropr. Man. Ther. 32, 20. doi: 10.1186/s12998-024-00541-4 38822395 PMC11143588

[B58] Fu S. Meng H. Inamdar S. Das B. Gupta H. Wang W. . (2021). Activation of TRPV4 by mechanical, osmotic or pharmaceutical stimulation is anti-inflammatory blocking IL-1β mediated articular cartilage matrix destruction. Osteoarthritis Cartilage 29, 89–99. doi: 10.1016/j.joca.2020.08.002 33395574 PMC7799379

[B59] Funabashi M. Breen A. C. De Carvalho D. Pagé I. Nougarou F. Descarreaux M. . (2022). Force distribution within spinal tissues during posterior to anterior spinal manipulative therapy: A secondary analysis. Front. Integr. Neurosci. 15. doi: 10.3389/fnint.2021.809372 35185486 PMC8855051

[B60] George S. Z. Fritz J. M. Silfies S. P. Schneider M. J. Beneciuk J. M. Lentz T. A. . (2021). Interventions for the management of acute and chronic low back pain: Revision 2021. J. Orthop. Sports Phys. Ther. 51, CPG1–CPG60. doi: 10.2519/jospt.2021.0304 34719942 PMC10508241

[B61] González‐Gómez L. Moral‐Munoz J. A. Rosales‐Tristancho A. Cuevas‐Moreno A. Cardellat‐González M. Rodríguez‐Domínguez Á. (2025). Exercise therapy versus manual therapy for the management of pain intensity, disability, and physical function in people with chronic low back pain: A systematic review with meta‐analysis and meta‐regression. Eur. J. Pain 29, e70090. doi: 10.1002/ejp.70090 40747709 PMC12314856

[B62] Goode A. P. Cleveland R. J. Kraus V. B. Taylor K. A. George S. Z. Schwartz T. A. . (2023). Biomarkers and longitudinal changes in lumbar spine degeneration and low back pain: The Johnston County Osteoarthritis Project. Osteoarthritis Cartilage 31, 809–818. doi: 10.1016/j.joca.2023.02.005 36804589 PMC10200763

[B63] Goodman L.-R. Dass R. Daniel E. Modarresi S. Carlesso L. Tang A. . (2025). Quantitative sensory testing and exercise-induced hypoalgesia protocols in low back pain: A scoping review. J. Pain 28, 104725. doi: 10.1016/j.jpain.2024.104725 39532209

[B64] Gouteron A. Tabard-Fougère A. Bourredjem A. Casillas J.-M. Armand S. Genevay S. (2021). The flexion relaxation phenomenon in nonspecific chronic low back pain: Prevalence, reproducibility and flexion–extension ratios. A systematic review and meta-analysis. Eur. Spine J. Off. Publ. Eur. Spine Soc Eur. Spinal Deform. Soc Eur. Sect. Cerv. Spine Res. Soc 31, 136–151. doi: 10.1007/s00586-021-06992-0 34553264

[B65] Guo S. Wang C. Xiao C. Gu Q. Long L. Wang X. . (2022). Role of the mechanosensitive piezo1 channel in intervertebral disc degeneration. Clin. Physiol. Funct. Imaging 43, 59–70. doi: 10.1111/cpf.12798 36400723

[B66] Han C. Chen J. Gao J. Wen C. Wen H. Yin X. . (2025). Kinematic and mechanical assessment of seated lumbar rotation manipulation: Force, velocity and orientation in three dimensions. Front. Bioeng. Biotechnol. 13. doi: 10.3389/fbioe.2025.1651760 41098615 PMC12518278

[B67] Harissa Z. Kim Y. Dicks A. R. Steward N. Guilak F. (2025). Skeletal dysplasia-causing mutations in TRPV4 alter the chondrocyte transcriptomic response to mechanical loading. Am. J. Physiol. Cell Physiol. 328, C1135–C1149. doi: 10.1152/ajpcell.01066.2024 40019039 PMC12288050

[B68] He J. Huang S. Li Y. Wang Y. Yan P. Hu O. . (2026). Collagenesis orchestrates mechanoadaptive development and homeostasis of intervertebral discs. Cell Rep. 45, 117076. doi: 10.1016/j.celrep.2026.117076 41818243

[B69] Hirata Y. Nomura K. Kato D. Tachibana Y. Niikura T. Uchiyama K. . (2022). A Piezo1/KLF15/IL-6 axis mediates immobilization-induced muscle atrophy. J. Clin. Invest. 132, 1–13. doi: 10.1172/jci154611 35290243 PMC9159676

[B70] Hodson N. W. Patel S. Richardson S. M. Hoyland J. A. Gilbert H. T. J. (2018). Degenerate intervertebral disc‐like pH induces a catabolic mechanoresponse in human nucleus pulposus cells. JOR Spine 1, e1004. doi: 10.1002/jsp2.1004 31463436 PMC6711490

[B71] Hu B. Lv X. Wei L. Wang Y. Zheng G. Yang C. . (2022). Sensory nerve maintains intervertebral disc extracellular matrix homeostasis via CGRP/CHSY1 axis. Adv. Sci. (Weinh) 9, 2202620. doi: 10.1002/advs.202202620 36047655 PMC9596848

[B72] Hu J. Zhu Y. Pang Z. Li X. Zhang H. Li X. . (2025). Low hydrostatic pressure promotes functional homeostasis of nucleus pulposus cells through the TRPV4/CRT/FA complex axis. Front. Med. 12. doi: 10.3389/fmed.2025.1531907 39944489 PMC11813897

[B73] Javaid H. Rehman S. Kashif M. Bashir M. Zia W. (2025). The effects of joint mobilization and myofascial release on muscle thickness in non-specific low back pain: A randomized clinical trial. J. Clin. Med. 14, 2830. doi: 10.3390/jcm14082830 40283659 PMC12028056

[B74] Jenks A. de Zoete A. van Tulder M. Rubinstein S. M. International IPD-SMT group (2022). Spinal manipulative therapy in older adults with chronic low back pain: An individual participant data meta-analysis. Eur. Spine J. Off. Publ. Eur. Spine Soc Eur. Spinal Deform. Soc Eur. Sect. Cerv. Spine Res. Soc 31, 1821–1845. doi: 10.1007/s00586-022-07210-1 35633383

[B75] Ji C. McCulloch C. A. (2020). TRPV4 integrates matrix mechanosensing with Ca2+ signaling to regulate extracellular matrix remodeling. FEBS J. 288, 5867–5887. doi: 10.1111/febs.15665 33300268

[B76] Jun P. Pagé I. Vette A. Kawchuk G. (2020). Potential mechanisms for lumbar spinal stiffness change following spinal manipulative therapy: A scoping review. Chiropr. Man. Ther. 28, 15. doi: 10.1186/s12998-020-00304-x 32293493 PMC7087370

[B77] Kameda T. Zvick J. Vuk M. Sadowska A. Tam W. K. Leung V. Y. . (2019). Expression and activity of TRPA1 and TRPV1 in the intervertebral disc: Association with inflammation and matrix remodeling. Int. J. Mol. Sci. 20, 1767. doi: 10.3390/ijms20071767 30974795 PMC6480240

[B78] Kanda Y. Yurube T. Morita Y. Takeoka Y. Kurakawa T. Tsujimoto R. . (2020). Delayed notochordal cell disappearance through integrin α5β1 mechanotransduction during ex‐vivo dynamic loading‐induced intervertebral disc degeneration. J. Orthop. Res. Off. Publ. Orthop. Res. Soc 39, 1933–1944. doi: 10.1002/jor.24883 33049071

[B79] Katz J. N. Zimmerman Z. E. Mass H. Makhni M. C. (2022). Diagnosis and management of lumbar spinal stenosis. JAMA 327, 1688. doi: 10.1001/jama.2022.5921 35503342

[B80] Ke W. Liao Z. Liang H. Tong B. Song Y. Li G. . (2023a). Stiff substrate induces nucleus pulposus cell ferroptosis via YAP and N‐cadherin mediated mechanotransduction. Adv. Healthcare Mater. 12, 2300458. doi: 10.1002/adhm.202300458 37022980

[B81] Ke W. Wang B. Liao Z. Song Y. Li G. Ma L. . (2023b). Matrix stiffness induces Drp1-mediated mitochondrial fission through Piezo1 mechanotransduction in human intervertebral disc degeneration. J. Transl. Med. 21, 711. doi: 10.1186/s12967-023-04590-w 37817199 PMC10563269

[B82] Ke W. Xu H. Zhang C. Liao Z. Liang H. Tong B. . (2025). An overview of mechanical microenvironment and mechanotransduction in intervertebral disc degeneration. Exp. Mol. Med. 57, 2157–2168. doi: 10.1038/s12276-025-01546-6 41034524 PMC12586686

[B83] Kibble M. J. Ferreira M. J. S. Usta Y. H. van den Akker G. G. H. Moxon S. R. Baird P. . (2025). Suspension bioprinted whole intervertebral disc analogues enable regional stiffness- and hypoxia-regulated matrix secretion by primary human nucleus pulposus and annulus fibrosus cells. Acta Biomater. 200, 378–389. doi: 10.1016/j.actbio.2025.05.015 40339969

[B84] Kim C. H. Chung C. K. Choi Y. Lee J. Yang S. H. Lee C. H. . (2021a). Direct medical costs after surgical or nonsurgical treatment for degenerative lumbar spinal disease: A nationwide matched cohort study with a 10-year follow-up. PloS One 16, e0260460. doi: 10.1371/journal.pone.0260460 34852015 PMC8635587

[B85] Kim M. K. Ramachandran R. Séguin C. A. (2021b). Spatiotemporal and functional characterisation of transient receptor potential vanilloid 4 (TRPV4) in the murine intervertebral disc. Eur. Cells Mater. 41, 194–203. doi: 10.22203/ecm.v041a14 33620083

[B86] Kim K. Yan D. Bauer B. A. Choi J.-C. Wang Z. Jung J. E. . (2026). Nonsurgical integrative treatments for symptomatic degenerative lumbar spondylolisthesis: A multinational randomized controlled clinical trial. Mayo Clin. Proc. 101, 955–962. doi: 10.1016/j.mayocp.2025.05.030 41176728

[B87] Knezevic N. N. Candido K. D. Vlaeyen J. W. S. Van Zundert J. Cohen S. P. (2021). Low back pain. Lancet 398, 78–92. doi: 10.1016/s0140-6736(21)00733-9 34115979

[B88] Köhli P. Schönnagel L. Hambrecht J. Zhu J. Chiapparelli E. Güven A. E. . (2024). The relationship between paraspinal muscle atrophy and degenerative lumbar spondylolisthesis at the L4/5 level. Spine J. Off. J. N. Am. Spine Soc 24, 1396–1406. doi: 10.1016/j.spinee.2024.03.016 38570036

[B89] Koppenhaver S. L. Weaver A. M. Randall T. L. Hollins R. J. Young B. A. Hebert J. J. . (2021). Effect of dry needling on lumbar muscle stiffness in patients with low back pain: A double blind, randomized controlled trial using shear wave elastography. J. Man. Manip. Ther. 30, 154–169. doi: 10.1080/10669817.2021.1977069 34525901 PMC9255226

[B90] Krupkova O. Aterini B. Rahal N. Schulze E. Darwiche S. Ehrbar M. . (2026). A mechanically active nucleus pulposus-on-a-chip for studying mechanobiology and therapeutic strategies in intervertebral disc disease. Biofabrication 18, 015031. doi: 10.1088/1758-5090/ae3d85 41587538

[B91] Kruse R. Gudavalli M. White B. Rider S. Greenwood D. Rogers C. (2025). Ultrasound measurements of lumbar spinous process movement during flexion distraction manipulation: A preliminary descriptive cross-sectional study with healthy participants. Chiropr. Man. Ther. 33, 29. doi: 10.1186/s12998-025-00593-0 40713659 PMC12291367

[B92] Langenfeld A. Baechler M. Swanenburg J. Mühlemann M. Nyirö L. Streuli D. . (2025). Systematic review on biomechanical effects of high-velocity, low amplitude spinal manipulation. PloS One 20, e0328048. doi: 10.1371/journal.pone.0328048 40680063 PMC12273944

[B93] Lee P. R. Ha T. Choi H. Lee S. E. Kim C. Hong G. (2024). Piezo1 mediates mechanical signals in TRPV1‐positive nociceptors in mice. Acta Physiol. (Oxf. Engl.) 240, e14236. doi: 10.1111/apha.14236 39324481

[B94] Lee W. Nims R. J. Savadipour A. Zhang Q. Leddy H. A. Liu F. . (2021). Inflammatory signaling sensitizes Piezo1 mechanotransduction in articular chondrocytes as a pathogenic feed-forward mechanism in osteoarthritis. Proc. Natl. Acad. Sci. U.S.A. 118, e2001611118. doi: 10.1073/pnas.2001611118 33758095 PMC8020656

[B95] Lei L. Wen Z. Cao M. Zhang H. Ling S. K.-K. Fu B. S.-C. . (2024). The emerging role of Piezo1 in the musculoskeletal system and disease. Theranostics 14, 3963–3983. doi: 10.7150/thno.96959 38994033 PMC11234281

[B96] Lei L. Zhang Q. Du M. Li L. (2025). Mechanoregulation of cell fate by low-intensity pulsed ultrasound: Mechanisms and advances in regenerative medicine. Bio Integration 6, 1–19. doi: 10.15212/bioi-2025-0049

[B97] Li F. Chen M. Zhang M. Chen S. Qu M. He S. . (2025). Targeting Piezo1 channel to alleviate intervertebral disc degeneration. J. Orthop. Transl. 51, 145–158. doi: 10.1016/j.jot.2025.01.006 40129609 PMC11930658

[B98] Li G. Feng C. Huang H. Deng J. Yang F. Chen R. (2026). The role of Piezo 1 in the study of intervertebral disc degeneration: Phenotype, mechanism and treatment. Orthop. Surg. 18, 897–914. doi: 10.1111/os.70291 41942106 PMC13139052

[B99] Li P. Liu C. Qian L. Zheng Z. Li C. Lian Z. . (2021). miR‐10396b‐3p inhibits mechanical stress‐induced ligamentum flavum hypertrophy by targeting IL‐11. FASEB J. Off. Publ. Fed. Am. Soc Exp. Biol. 35, e21676. doi: 10.1096/fj.202100169rr 34042220

[B100] Li L. Shen T. Li Y.-K. (2017). A finite element analysis of stress distribution and disk displacement in response to lumbar rotation manipulation in the sitting and side-lying positions. J. Manipulative Physiol. Ther. 40, 580–586. doi: 10.1016/j.jmpt.2017.07.006 29187309

[B101] Li H. Tang Y. Liu Z. Chen K. Zhang K. Hu S. . (2024). Lumbar instability remodels cartilage endplate to induce intervertebral disc degeneration by recruiting osteoclasts via Hippo-CCL3 signaling. Bone Res. 12, 34. doi: 10.1038/s41413-024-00331-x 38816384 PMC11139958

[B102] Liebsch C. Wilke H.-J. (2025). The intradiscal pressure of the lumbar spine is affected by intervertebral disc degeneration, age, level, and motion direction: evaluation of an *in vitro* database comprising 107 specimens. Spine J. Off. J. N. Am. Spine Soc 25, 1276–1287. doi: 10.1016/j.spinee.2025.01.024 39894272

[B103] Lin Z. Xu G. Lu X. Wang H. Lu F. Xia X. . (2024). Piezo1 exacerbates inflammation‐induced cartilaginous endplate degeneration by activating mitochondrial fission via the Ca2+/CaMKII/Drp1 axis. Aging Cell 24, e14440. doi: 10.1111/acel.14440 39610146 PMC11984661

[B104] Lisi A. J. O’Neill C. W. Lindsey D. P. Cooperstein R. Cooperstein E. Zucherman J. F. (2006). Measurement of *in vivo* lumbar intervertebral disc pressure during spinal manipulation: a feasibility study. J. Appl. Biomech. 22, 234–239. doi: 10.1123/jab.22.3.234 17215555

[B105] Liu C. Gao X. Lou J. Li H. Chen Y. Chen M. . (2023). Aberrant mechanical loading induces annulus fibrosus cells apoptosis in intervertebral disc degeneration via mechanosensitive ion channel Piezo1. Arthritis Res. Ther. 25, 117. doi: 10.1186/s13075-023-03093-9 37420255 PMC10327399

[B106] Liu C. Li P. Ao X. Lian Z. Liu J. Li C. . (2022). Clusterin negatively modulates mechanical stress-mediated ligamentum flavum hypertrophy through TGF-β1 signaling. Exp. Mol. Med. 54, 1549–1562. doi: 10.1038/s12276-022-00849-2 36131026 PMC9534863

[B107] Liu S. Shi Q. Da W.-W. Xue C.-C. Chen L. Li Y.-N. . (2024b). Association between paraspinal muscle parameters and single-segment degenerative lumbar spondylolisthesis. Spine 50, 841–848. doi: 10.1097/brs.0000000000005140 39192738 PMC12101887

[B108] Liu Z. Wen H. Zhu Y. Zhao B. Kong Q. Chen J. . (2021). Short-term effect of lumbar traction on intervertebral discs in patients with low back pain: correlation between the T2 value and ODI/VAS score. Cartilage 13, 414S–423S. doi: 10.1177/1947603521996793 33622056 PMC8808794

[B109] Liu K. Zhao T. Zhang Y. Chen L. Zhang H. Xu X. . (2024a). Shear wave elastography based analysis of changes in fascial and muscle stiffness in patients with chronic non-specific low back pain. Front. Bioeng. Biotechnol. 12. doi: 10.3389/fbioe.2024.1476396 39619623 PMC11604440

[B110] Lyu B. Lin J. Chen L. Wang Y. (2026). The emerging role of TRPV4 in skeletal biology: mechanotransduction, mutation, and therapeutic potential. Cell. Calcium 134, 103108. doi: 10.1016/j.ceca.2026.103108 41529958

[B111] Maiers M. J. Sundin A. R. Oster R. J. Kreul S. Malone Q. Passmore S. R. (2025). Contextual factors related to aging determine force-based manipulation dosage: a prospective cross-sectional study. Chiropr. Man. Ther. 33, 20. doi: 10.1186/s12998-025-00584-1 40399997 PMC12093877

[B112] Maigne J.-Y. Guillon F. (2000). Highlighting of intervertebral movements and variations of intradiskal pressure during lumbar spine manipulation: a feasibility study. J. Manipulative Physiol. Ther. 23, 531–535. doi: 10.1067/mmt.2000.109679 11050609

[B113] Mark Kim M. K. Lawrence M. Quinonez D. Brooks C. Ramachandran R. Séguin C. A. (2024). Transient receptor potential vanilloid 4 regulates extracellular matrix composition and mediates load-induced intervertebral disc degeneration in a mouse model. Osteoarthritis Cartilage 32, 881–894. doi: 10.1016/j.joca.2024.04.001 38604493

[B114] Matsuo T. Takeoka Y. Yurube T. Tsujimoto T. Kanda Y. Miyazaki K. . (2025). Transient receptor potential vanilloid 4 knockdown decreases extracellular matrix synthesis via autophagy suppression in the rat intervertebral disc. Jor Spine 8, e70046. doi: 10.1002/jsp2.70046 39963549 PMC11832302

[B115] Matyas J. R. Klein C. Ponjevic D. Duncan N. A. Kawchuk G. N. (2021). Repetitive *in vivo* manual loading of the spine elicits cellular responses in porcine annuli fibrosi. PloS One 16, e0248104. doi: 10.1371/journal.pone.0248104 33755684 PMC7987143

[B116] Matziolis G. Bergner L. Hawi H. Bauer L. Woiczinski M. Strube P. . (2025). A rig for *in vitro* testing of the lumbar spine and pelvis simulating posterior, anterior and oblique trunk muscles. Sci. Rep. 15, 9377. doi: 10.1038/s41598-025-93599-w 40102515 PMC11920589

[B117] McGrath K. A. Lee J. Thompson N. R. Kanasz J. Steinmetz M. P. (2022). Identifying the relationship between lumbar sacralization and adjacent ligamentous anatomy in patients with Bertolotti syndrome and healthy controls. J. Neurosurg. Spine 37, 200–207. doi: 10.3171/2021.12.spine211116 35148504

[B118] Morales N. P. Loahachanwanich W. Korwutthikulrangsri E. Ruangchainikom M. Sutipornpalangkul W. (2024). Lipid peroxidation, reduced glutathione, and glutathione peroxidase levels in intervertebral discs of patients with lumbar degenerative disc disease. Med. Sci. Monit. Int. Med. J. Exp. Clin. Res. 30, e944335. doi: 10.12659/msm.944335 38783538 PMC11131429

[B119] Murthy S. E. (2023). Deciphering mechanically activated ion channels at the single-channel level in dorsal root ganglion neurons. J. Gen. Physiol. 155, e202213099. doi: 10.1085/jgp.202213099 37102984 PMC10140383

[B120] Murthy S. E. Loud M. C. Daou I. Marshall K. L. Schwaller F. Kühnemund J. . (2018). The mechanosensitive ion channel Piezo2 mediates sensitivity to mechanical pain in mice. Sci. Transl. Med. 10, eaat9897. doi: 10.1126/scitranslmed.aat9897 30305457 PMC6709986

[B121] Naha A. Driscoll T. P. (2023). Fibronectin sensitizes activation of contractility, YAP, and NF‐κB in nucleus pulposus cells. J. Orthop. Res. Off. Publ. Orthop. Res. Soc 42, 434–442. doi: 10.1002/jor.25670 37525423

[B122] Nieuwstraten J. Riester R. Hofmann U. K. Guilak F. Danalache M. (2025). Matrix metalloproteinases accelerate pericellular matrix breakdown and disrupt mechanotransduction in osteoarthritis. Acta Biomater. 195, 73–82. doi: 10.1016/j.actbio.2025.02.034 39956307

[B123] Nijs J. Kosek E. Chiarotto A. Cook C. Danneels L. A. Fernández-de-las-Peñas C. . (2024). Nociceptive, neuropathic, or nociplastic low back pain? The low back pain phenotyping (BACPAP) consortium’s international and multidisciplinary consensus recommendations. Lancet Rheumatol. 6, e178–e188. doi: 10.1016/s2665-9913(23)00324-7 38310923

[B124] Nim C. Aspinall S. L. Cook C. E. Corrêa L. A. Donaldson M. Downie A. S. . (2025). The effectiveness of spinal manipulative therapy in treating spinal pain does not depend on the application procedures: a systematic review and network meta-analysis. J. Orthop. Sports Phys. Ther. 55, 109–122. doi: 10.2519/jospt.2025.12707 39869665

[B125] Nim C. G. Kawchuk G. N. Schiøttz-Christensen B. O’Neill S. (2020). The effect on clinical outcomes when targeting spinal manipulation at stiffness or pain sensitivity: a randomized trial. Sci. Rep. 10, 14615. doi: 10.1038/s41598-020-71557-y 32884045 PMC7471938

[B126] Nim C. G. Kawchuk G. N. Schiøttz-Christensen B. O’Neill S. (2021). Changes in pain sensitivity and spinal stiffness in relation to responder status following spinal manipulative therapy in chronic low back pain: a secondary explorative analysis of a randomized trial. BMC Musculoskelet. Disord. 22, 23. doi: 10.1186/s12891-020-03873-3 33407345 PMC7786943

[B127] Nims R. Palmer D. R. Kassab J. Zhang B. Guilak F. (2024). The chondrocyte “mechanome”: activation of the mechanosensitive ion channels TRPV4 and PIEZO1 drives unique transcriptional signatures. FASEB J. Off. Publ. Fed. Am. Soc Exp. Biol. 38, e23778. doi: 10.1096/fj.202400883r 38959010 PMC11327906

[B128] Noonan A. M. Brown S. H. M. (2021). Paraspinal muscle pathophysiology associated with low back pain and spine degenerative disorders. Jor Spine 4, e1171. doi: 10.1002/jsp2.1171 34611593 PMC8479522

[B129] Nummenmaa E. Hämäläinen M. Pemmari A. Moilanen L. J. Tuure L. Nieminen R. M. . (2020). Transient receptor potential ankyrin 1 (TRPA1) is involved in upregulating interleukin-6 expression in osteoarthritic chondrocyte models. Int. J. Mol. Sci. 22, 87. doi: 10.3390/ijms22010087 33374841 PMC7794684

[B130] Nyirö L. Dörig M. Suter M. Connolly L. Vogel N. Stadler C. . (2025). The impact of spinal manipulation on lumbar proprioception and its link to pain relief: a randomized controlled trial. Sci. Rep. 15, 42136. doi: 10.1038/s41598-025-25985-3 41298593 PMC12658151

[B131] Nyirö L. Gorrell L. M. Cecchini V. Menon C. Elgendi M. Schweinhardt P. (2024). Variability and repeatability of spinal manipulation force–time characteristics in thoracic spinal manipulation on a manikin. Chiropr. Man. Ther. 32, 33. doi: 10.1186/s12998-024-00551-2 39529165 PMC11552221

[B132] Obeidat A. M. Wood M. J. Adamczyk N. S. Ishihara S. Li J. Wang L. . (2023). Piezo2 expressing nociceptors mediate mechanical sensitization in experimental osteoarthritis. Nat. Commun. 14, 2479. doi: 10.1038/s41467-023-38241-x 37120427 PMC10148822

[B133] Owen P. J. Miller C. T. Mundell N. L. Verswijveren S. J. J. M. Tagliaferri S. D. Brisby H. . (2019). Which specific modes of exercise training are most effective for treating low back pain? Network meta-analysis. Br. J. Sports Med. 54, 1279–1287. doi: 10.1136/bjsports-2019-100886 31666220 PMC7588406

[B134] Peng Y. Du J. Günther S. Guo X. Wang S. Schneider A. . (2022). Mechano-signaling via Piezo1 prevents activation and p53-mediated senescence of muscle stem cells. Redox Biol. 52, 102309. doi: 10.1016/j.redox.2022.102309 35395625 PMC9005960

[B135] Peng F. Sun M. Jing X. Chen F. Cao T. Li Z. . (2025). Piezo1 promotes intervertebral disc degeneration through the Ca2+/F-actin/Yap signaling axis. Mol. Med. (Camb. Mass,) 31, 90. doi: 10.1186/s10020-025-01147-z 40057686 PMC11889814

[B136] Pirri C. Pirri N. Macchi V. Porzionato A. De Caro R. Stecco C. (2024). Ultrasound imaging of thoracolumbar fascia: a systematic review. Med. (Mex.) 60, 1090. doi: 10.3390/medicina60071090 39064519 PMC11279050

[B137] Qin L. He T. Chen S. Yang D. Yi W. Cao H. . (2021). Roles of mechanosensitive channel Piezo1/2 proteins in skeleton and other tissues. Bone Res. 9, 44. doi: 10.1038/s41413-021-00168-8 34667178 PMC8526690

[B138] Rangwalla K. Filley A. El Naga A. Gendelberg D. Baldwin A. Maziad A. . (2023). Degenerative lumbar spondylolisthesis: review of current classifications and proposal of a novel classification system. Eur. Spine J. 33, 1762–1772. doi: 10.1007/s00586-023-07818-x 37543967

[B139] Rashidi N. Harasymowicz N. S. Savadipour A. Steward N. Tang R. Oswald S. . (2025). PIEZO1-mediated mechanotransduction regulates collagen synthesis on nanostructured 2D and 3D models of fibrosis. Acta Biomater. 193, 242–254. doi: 10.1016/j.actbio.2024.12.034 39675497 PMC13276715

[B140] Ren X. Zhuang H. Li B. Jiang F. Zhang Y. Zhou P. (2023). Gsmtx4 alleviated osteoarthritis through Piezo1/Calcineurin/NFAT1 signaling axis under excessive mechanical strain. Int. J. Mol. Sci. 24, 4022. doi: 10.3390/ijms24044022 36835440 PMC9961447

[B141] Roh J. Hwang S.-M. Lee S.-H. Lee K. Kim Y. H. Park C.-K. (2020). Functional expression of Piezo1 in dorsal root ganglion (DRG) neurons. Int. J. Mol. Sci. 21, 3834. doi: 10.3390/ijms21113834 32481599 PMC7313462

[B142] Ruffilli A. Viroli G. Neri S. Traversari M. Barile F. Manzetti M. . (2023). Mechanobiology of the human intervertebral disc: systematic review of the literature and future perspectives. Int. J. Mol. Sci. 24, 2728. doi: 10.3390/ijms24032728 36769050 PMC9917554

[B143] Ruzich J. J. Klopper M. Dohrmann C. M. Rhon D. I. Young J. L. (2024). How reproducible are manual therapy interventions in trials for low back pain? A scoping review. J. Orthop. Sports Phys. Ther. 54, 248–257. doi: 10.2519/jospt.2024.12201 38284379

[B144] Sadowska A. Altinay B. Hitzl W. Ferguson S. J. Wuertz-Kozak K. (2020). Hypo-osmotic loading induces expression of IL-6 in nucleus pulposus cells of the intervertebral disc independent of TRPV4 and TRPM7. Front. Pharmacol. 11. doi: 10.3389/fphar.2020.00952 32714187 PMC7341822

[B145] Sadowska A. Hitzl W. Karol A. Jaszczuk P. Cherif H. Haglund L. . (2019). Differential regulation of TRP channel gene and protein expression by intervertebral disc degeneration and back pain. Sci. Rep. 9, 18889. doi: 10.1038/s41598-019-55212-9 31827137 PMC6906425

[B146] Salzer E. Schmitz T. C. Mouser V. H. Vernengo A. Gantenbein B. Jansen J. U. . (2023). Ex vivo intervertebral disc cultures: degeneration-induction methods and their implications for clinical translation. Eur. Cells Mater. 45, 88–112. doi: 10.22203/ecm.v045a07 36989118

[B147] Samuel A. M. Moore H. G. Cunningham M. E. (2017). Treatment for degenerative lumbar spondylolisthesis: current concepts and new evidence. Curr. Rev. Musculoskelet. Med. 10, 521–529. doi: 10.1007/s12178-017-9442-3 28994028 PMC5685964

[B148] Sánchez-Vera M. A. Serrano Diaz D. F. Canty Casallas J. K. Bonilla Rodríguez P. A. Schleip R. Alfonso-Mora M. L. (2025). Effect of crossed-hands myofascial induction on thoracolumbar fascia stiffness, thickness, and alignment. J. Bodyw. Mov. Ther. 43, 338–343. doi: 10.1016/j.jbmt.2025.05.017 40483145

[B149] Sao K. Risbud M. V. (2025). SDC4 drives fibrotic remodeling of the intervertebral disc under altered spinal loading. Cell Death Dis. 16, 678. doi: 10.1038/s41419-025-08002-3 41053113 PMC12500954

[B150] Savadipour A. Palmer D. Ely E. V. Collins K. H. Garcia-Castorena J. M. Harissa Z. . (2023). The role of PIEZO ion channels in the musculoskeletal system. Am. J. Physiol. Cell Physiol. 324, C728–C740. doi: 10.1152/ajpcell.00544.2022 36717101 PMC10027092

[B151] Savage N. J. George K. Gibson E. Taylor K. (2025). Evaluation of lumbar segmental motion using ultrasound imaging following common joint mobilization techniques. J. Man. Manip. Ther. 33, 343–355. doi: 10.1080/10669817.2025.2470464 40067264 PMC12281658

[B152] Shi S. Kang X.-J. Zhou Z. He Z.-M. Zheng S. He S.-S. (2022). Excessive mechanical stress-induced intervertebral disc degeneration is related to Piezo1 overexpression triggering the imbalance of autophagy/apoptosis in human nucleus pulpous. Arthritis Res. Ther. 24, 119. doi: 10.1186/s13075-022-02804-y 35606793 PMC9125856

[B153] Shitozawa H. Nakamichi R. Yoshida A. Ueda M. Saito T. Uotani K. . (2026). Mechanosensitive ion channel PIEZO1 suppresses BMP2‐induced ossification of the annulus fibrosus cells. JOR Spine 9, e70168. doi: 10.1002/jsp2.70168 41783545 PMC12954436

[B154] Shraim M. A. Massé‐Alarie H. Farrell M. J. Cavaleri R. Loggia M. L. Hodges P. W. (2024). Neuroinflammatory activation in sensory and motor regions of the cortex is related to sensorimotor function in individuals with low back pain maintained by nociplastic mechanisms: A preliminary proof‐of‐concept study. Eur. J. Pain 28, 1607–1626. doi: 10.1002/ejp.2313 39007713

[B155] Silwal P. Nguyen-Thai A. M. Alexander P. G. Sowa G. A. Vo N. V. Lee J. Y. (2024). Cellular and molecular mechanisms of hypertrophy of ligamentum flavum. Biomolecules 14, 1277. doi: 10.3390/biom14101277 39456209 PMC11506588

[B156] Sinhorim L. Amorim M. S. Ortiz M. E. Bittencourt E. B. Bianco G. da Silva F. C. . (2021). Potential nociceptive role of the thoracolumbar fascia: A scope review involving *in vivo* and ex vivo studies. J. Clin. Med. 10, 4342. doi: 10.3390/jcm10194342 34640360 PMC8509394

[B157] Soubrier A. Kasper H. Vonlanthen N. Jonkers I. Grad S. (2025). Short‐term dynamic unloading of bovine tail discs in culture partially mitigates induced degeneration after one‐strike trigger. JOR Spine 8, e70092. doi: 10.1002/jsp2.70092 40671965 PMC12266091

[B158] Sun Y. Leng P. Song M. Li D. Guo P. Xu X. . (2020). Piezo1 activates the NLRP3 inflammasome in nucleus pulposus cell-mediated by Ca2+/NF-κB pathway. Int. Immunopharmacol. 85, 106681. doi: 10.1016/j.intimp.2020.106681 32526681

[B159] Sun C. Ma Q. Yin J. Zhang H. Liu X. (2021a). WISP-1 induced by mechanical stress contributes to fibrosis and hypertrophy of the ligamentum flavum through Hedgehog-Gli1 signaling. Exp. Mol. Med. 53, 1068–1079. doi: 10.1038/s12276-021-00636-5 34158608 PMC8257797

[B160] Sun H. Wu Z. Zhang Y. Liu C. Gao D. Zhao Q. . (2026). Excessive compression induces cartilage endplate degeneration via the Piezo1/NAT10/mTOR signaling axis. Osteoarthritis Cartilage 34, 550–562. doi: 10.1016/j.joca.2025.10.014 41175920

[B161] Sun Z. Zheng X. Li S. Zeng B. Yang J. Ling Z. . (2021b). Single impact injury of vertebral endplates without structural disruption, initiates disc degeneration through Piezo1 mediated inflammation and metabolism dysfunction. Spine 47, E203–E213. doi: 10.1097/brs.0000000000004203 34431832 PMC8815838

[B162] Swahn H. Mertens J. Olmer M. Myers K. Mondala T. S. Natarajan P. . (2024). Shared and compartment‐specific processes in nucleus pulposus and annulus fibrosus during intervertebral disc degeneration. Adv. Sci. 11, 2309032. doi: 10.1002/advs.202309032 38403470 PMC11077672

[B163] Szczot M. Liljencrantz J. Ghitani N. Barik A. Lam R. Thompson J. H. . (2018). PIEZO2 mediates injury-induced tactile pain in mice and humans. Sci. Transl. Med. 10, eaat9892. doi: 10.1126/scitranslmed.aat9892 30305456 PMC6875774

[B164] Taiji R. Kang J. D. Mizuno S. (2024). Cellular behavior and extracellular matrix turnover in bovine annulus fibrosus cells under hydrostatic pressure and deviatoric strain. J. Orthop. Res. Off. Publ. Orthop. Res. Soc 42, 1326–1334. doi: 10.1002/jor.25779 38153697

[B165] Takeoka Y. Paladugu P. Kang J. D. Mizuno S. (2021). Augmented chondroitin sulfate proteoglycan has therapeutic potential for intervertebral disc degeneration by stimulating anabolic turnover in bovine nucleus pulposus cells under changes in hydrostatic pressure. Int. J. Mol. Sci. 22, 6015. doi: 10.3390/ijms22116015 34199496 PMC8199579

[B166] Tong T. Wang F. Miao D. Wang L. (2023). Piezo1 involves in intracellular osteogenic transformation signal to promote the ossification of ligamentum flavum. Biochem. Biophys. Res. Commun. 662, 114–118. doi: 10.1016/j.bbrc.2023.04.025 37104881

[B167] Trevisani M. Siemens J. Materazzi S. Bautista D. M. Nassini R. Campi B. . (2007). 4-hydroxynonenal, an endogenous aldehyde, causes pain and neurogenic inflammation through activation of the irritant receptor TRPA1. Proc. Natl. Acad. Sci. U.S.A. 104, 13519–13524. doi: 10.1073/pnas.0705923104 17684094 PMC1948902

[B168] Tseng M.-C. Lim J. Chu Y.-C. Chen C.-W. Feng C.-K. Wang J.-L. (2021). Dynamic pressure stimulation upregulates collagen II and aggrecan in nucleus pulposus cells through calcium signaling. Spine 47, 1111–1119. doi: 10.1097/brs.0000000000004286 34812197

[B169] Vadala G. Russo F. Han I.-H. Jain A. Tavakoli J. AO Spine Knowledge Forum Degenerative (2026). The biomechanical landscape of lumbar disc herniation: Mechanobiological insights into injury and regeneration. Neurospine 23, 159–175. doi: 10.14245/ns.2551668.834 41666869 PMC12890390

[B170] Vanti C. Ferrari S. Guccione A. A. Pillastrini P. (2021). Lumbar spondylolisthesis: State of the art on assessment and conservative treatment. Arch. Physiother. 11, 19. doi: 10.1186/s40945-021-00113-2 34372944 PMC8351422

[B171] Vieira F. Kang J. Ferreira L. Mizuno S. (2021). Hydrostatic pressure mimicking diurnal spinal movements maintains anabolic turnover in bovine nucleus pulposus cells *in vitro*. Eur. Cells Mater. 42, 246–263. doi: 10.22203/ecm.v042a18 34618349

[B172] Walter B. A. Purmessur D. Moon A. Occhiogrosso J. Laudier D. M. Hecht A. C. . (2016). Reduced tissue osmolarity increases TRPV4 expression and pro-inflammatory cytokines in intervertebral disc cells. Eur. Cells Mater. 32, 123–136. doi: 10.22203/ecm.v032a08 27434269 PMC5072776

[B173] Wang Y. Bai B. Hu Y. Wang H. Liu N. Li Y. . (2021b). Hydrostatic pressure modulates intervertebral disc cell survival and extracellular matrix homeostasis via regulating Hippo-YAP/TAZ pathway. Stem Cells Int. 2021, 1–17. doi: 10.1155/2021/5626487 34221023 PMC8221882

[B174] Wang K. Deng Z. Chen X. Shao J. Qiu L. Jiang C. . (2023c). The role of multifidus in the biomechanics of lumbar spine: A musculoskeletal modeling study. Bioengineering 10, 67. doi: 10.3390/bioengineering10010067 36671639 PMC9854514

[B175] Wang Y. X. J. Káplár Z. Deng M. Leung J. C. S. (2017). Lumbar degenerative spondylolisthesis epidemiology: A systematic review with a focus on gender-specific and age-specific prevalence. J. Orthop. Transl. 11, 39–52. doi: 10.1016/j.jot.2016.11.001 29662768 PMC5866399

[B176] Wang B. Ke W. Wang K. Li G. Ma L. Lu S. . (2021a). Mechanosensitive ion channel Piezo1 activated by matrix stiffness regulates oxidative stress‐induced senescence and apoptosis in human intervertebral disc degeneration. Oxid. Med. Cell. Longevity 2021, 8884922. doi: 10.1155/2021/8884922 33628392 PMC7889339

[B177] Wang D. Li Z. Huang W. Cao S. Xie L. Chen Y. . (2023a). Single-cell transcriptomics reveals heterogeneity and intercellular crosstalk in human intervertebral disc degeneration. iScience 26, 106692. doi: 10.1016/j.isci.2023.106692 37216089 PMC10192848

[B178] Wang S. Li W. Zhang P. Wang Z. Ma X. Liu C. . (2022). Mechanical overloading induces GPX4-regulated chondrocyte ferroptosis in osteoarthritis via Piezo1 channel facilitated calcium influx. J. Adv. Res. 41, 63–75. doi: 10.1016/j.jare.2022.01.004 36328754 PMC9637484

[B179] Wang H. Zhang W. Cai Y. Guo Q. Pan L. Chu G. . (2023b). Moderate mechanical stimulation antagonizes inflammation of annulus fibrosus cells through YAP‐mediated suppression of NF‐κB signaling. J. Orthop. Res. Off. Publ. Orthop. Res. Soc 41, 2667–2684. doi: 10.1002/jor.25596 37132373

[B180] Wang F. Zhang J. Feng W. Liu Q. Yang X. Zhang H. . (2018). Comparison of human lumbar disc pressure characteristics during simulated spinal manipulation vs. spinal mobilization. Mol. Med. Rep 18, 5709–5716. doi: 10.3892/mmr.2018.9591 30365136

[B181] Wenger L. E. Barrett D. R. Rhon D. I. Young J. L. (2023). Evaluating and characterizing the scope of care for interventions labeled as manual therapy in low back pain trials: A scoping review. Phys. Ther. 104, pzad178. doi: 10.1093/ptj/pzad178 38157290

[B182] Whedon J. M. Kizhakkeveettil A. Toler A. W. Bezdjian S. Rossi D. Uptmor S. . (2021). Initial choice of spinal manipulation reduces escalation of care for chronic low back pain among older Medicare beneficiaries. Spine 47, E142–E148. doi: 10.1097/brs.0000000000004118 34474443 PMC8581066

[B183] Widmer J. Fornaciari P. Senteler M. Roth T. Snedeker J. G. Farshad M. (2019). Kinematics of the spine under healthy and degenerative conditions: A systematic review. Ann. Biomed. Eng. 47, 1491–1522. doi: 10.1007/s10439-019-02252-x 30937563

[B184] Wirth B. Schweinhardt P. (2023). Personalized assessment and management of non‐specific low back pain. Eur. J. Pain 28, 181–198. doi: 10.1002/ejp.2190 37874300

[B185] Woo S.-H. Lukacs V. de Nooij J. C. Zaytseva D. Criddle C. R. Francisco A. . (2015). Piezo2 is the principal mechanotransduction channel for proprioception. Nat. Neurosci. 18, 1756–1762. doi: 10.1038/nn.4162 26551544 PMC4661126

[B186] Wu J. Chen Y. Liao Z. Liu H. Zhang S. Zhong D. . (2022). Self-amplifying loop of NF-κB and periostin initiated by PIEZO1 accelerates mechano-induced senescence of nucleus pulposus cells and intervertebral disc degeneration. Mol. Ther. J. Am. Soc Gene Ther. 30, 3241–3256. doi: 10.1016/j.ymthe.2022.05.021 35619555 PMC9552911

[B187] Xiang Z. Zhang P. Jia C. Xu R. Cao D. Xu Z. . (2024). Piezo1 channel exaggerates ferroptosis of nucleus pulposus cells by mediating mechanical stress-induced iron influx. Bone Res. 12, 20. doi: 10.1038/s41413-024-00317-9 38553442 PMC10980708

[B188] Xiaojun Z. Chen Q. Liu S. Liu B. (2025). *In vitro* evaluation of Verteporfin and exploration of TEAD palmitoylation inhibition in Piezo1–YAP/TAZ signaling and ECM remodeling. Sci. Rep. 15, 44048. doi: 10.1038/s41598-025-28960-0 41407746 PMC12715214

[B189] Xie Z. Feng J. Hibberd T. J. Chen B. N. Zhao Y. Zang K. . (2023). Piezo2 channels expressed by colon-innervating TRPV1-lineage neurons mediate visceral mechanical hypersensitivity. Neuron 111, 526–538.e4. doi: 10.1016/j.neuron.2022.11.015 36563677 PMC9957938

[B190] Xie W. Lückemeyer D. D. Qualls K. A. Prudente A. S. Berta T. Gu M. . (2025). Vascular motion in the dorsal root ganglion sensed by Piezo2 in sensory neurons triggers episodic pain. Neuron 113, 1774–1788.e5. doi: 10.1016/j.neuron.2025.03.006 40154477 PMC12140901

[B191] Xu H. Wei K. Ni J. Deng X. Wang Y. Xiang T. . (2025). Matrix stiffness regulates nucleus pulposus cell glycolysis by MRTF-A-dependent mechanotransduction. Bone Res. 13, 23. doi: 10.1038/s41413-025-00402-7 39952914 PMC11828926

[B192] Yabu A. Suzuki A. Hayashi K. Hori Y. Terai H. Orita K. . (2022). Periostin increased by mechanical stress upregulates interleukin‐6 expression in the ligamentum flavum. FASEB J. Off. Publ. Fed. Am. Soc Exp. Biol. 37, e22726. doi: 10.1096/fj.202200917rr 36583686

[B193] Ye J. Zhu J. Kuang K. Li Y. Su C. (2025). Transcriptomic insights into the anti-apoptotic effect of Tuina in rabbit model of intervertebral disc degeneration. J. Orthop. Surg. Res. 20, 1005. doi: 10.1186/s13018-025-06390-y 41254757 PMC12625494

[B194] Yu Y. Xu W. Li X. Zeng X. Su Z. Wang Q. . (2025). MRI radiomics based on paraspinal muscle for prediction postoperative outcomes in lumbar degenerative spondylolisthesis. Eur. Spine J. Off. Publ. Eur. Spine Soc Eur. Spinal Deform. Soc Eur. Sect. Cerv. Spine Res. Soc 34, 3820–3831. doi: 10.1007/s00586-025-08959-x 40455236

[B195] Zaworski K. Baj-Korpak J. Tokarska-Rodak M. Plażuk E. Dyrda A. Bialosky J. . (2025). Can pre-treatment verbal suggestions influence the short-term effects of spinal manipulation in young adults with chronic non-specific low back pain? A randomized controlled trial. Musculoskelet. Sci. Pract. 80, 103431. doi: 10.1016/j.msksp.2025.103431 41106172

[B196] Zhang W. Wang H. Yuan Z. Chu G. Sun H. Yu Z. . (2021). Moderate mechanical stimulation rescues degenerative annulus fibrosus by suppressing caveolin-1 mediated pro-inflammatory signaling pathway. Int. J. Biol. Sci. 17, 1395–1412. doi: 10.7150/ijbs.57774 33867854 PMC8040478

[B197] Zhao Q. Zhou H. Chi S. Wang Y. Wang J. Geng J. . (2018). Structure and mechanogating mechanism of the Piezo1 channel. Nature 554, 487–492. doi: 10.1038/nature25743 29469092

[B198] Zheng L. Cao Y. Ni S. Qi H. Ling Z. Xu X. . (2018). Ciliary parathyroid hormone signaling activates transforming growth factor-β to maintain intervertebral disc homeostasis during aging. Bone Res. 6, 21. doi: 10.1038/s41413-018-0022-y 30038820 PMC6050246

[B199] Zheng J. Zhang X. Zhang F. Zhao M. Wang L. Han T. . (2026). Hydrogels targeting mechanical–biological signaling transitions of interleukin-33 alleviates intervertebral-disc degeneration. Mater. Today Bio 36, 102726. doi: 10.1016/j.mtbio.2025.102726 41560790 PMC12813325

